# Ceria nanoparticles ameliorate white matter injury after intracerebral hemorrhage: microglia-astrocyte involvement in remyelination

**DOI:** 10.1186/s12974-021-02101-6

**Published:** 2021-02-15

**Authors:** Jingwei Zheng, Jia’nan Lu, Shuhao Mei, Haijian Wu, Zeyu Sun, Yuanjian Fang, Shenbin Xu, Xiaoyu Wang, Ligen Shi, Weilin Xu, Sheng Chen, Jun Yu, Feng Liang, Jianmin Zhang

**Affiliations:** 1 Department of Neurosurgery, Second Affiliated Hospital, School of Medicine, Zhejiang University, Hangzhou, Zhejiang China; 2 Brain Research Institute, Zhejiang University, Hangzhou, Zhejiang China; 3 Collaborative Innovation Center for Brain Science, Zhejiang University, Hangzhou, Zhejiang China

**Keywords:** Intracerebral hemorrhage, Reactive oxygen species, White matter injury, Microglia, Astrocyte

## Abstract

**Background:**

Intracerebral hemorrhage (ICH) can induce excessive accumulation of reactive oxygen species (ROS) that may subsequently cause severe white matter injury. The process of oligodendrocyte progenitor cell (OPC) differentiation is orchestrated by microglia and astrocytes, and ROS also drives the activation of microglia and astrocytes. In light of the potent ROS scavenging capacity of ceria nanoparticles (CeNP), we aimed to investigate whether treatment with CeNP ameliorates white matter injury by modulating ROS-induced microglial polarization and astrocyte alteration.

**Methods:**

ICH was induced in vivo by collagenase VII injection. Mice were administered with PLX3397 for depleting microglia. Primary microglia and astrocytes were used for in vitro experiments. Transmission electron microscopy analysis and immunostaining were performed to verify the positive effects of CeNP in remyelination and OPC differentiation. Flow cytometry, real-time polymerase chain reaction, immunofluorescence and western blotting were used to detect microglia polarization, astrocyte alteration, and the underlying molecular mechanisms.

**Results:**

CeNP treatment strongly inhibited ROS-induced NF-κB p65 translocation in both microglia and astrocytes, and significantly decreased the expression of M1 microglia and A1 astrocyte. Furthermore, we found that CeNP treatment promoted remyelination and OPC differentiation after ICH, and such effects were alleviated after microglial depletion. Interestingly, we also found that the number of mature oligodendrocytes was moderately increased in ICH + CeNP + PLX3397-treated mice compared to the ICH + vehicle + PLX3397 group. Therefore, astrocytes might participate in the pathophysiological process. The subsequent phagocytosis assay indicated that A1 astrocyte highly expressed C3, which could bind with microglia C3aR and hinder microglial engulfment of myelin debris. This result further replenished the feedback mechanism from astrocytes to microglia.

**Conclusion:**

The present study reveals a new mechanism in white matter injury after ICH: ICH induces M1 microglia and A1 astrocyte through ROS-induced NF-κB p65 translocation that hinders OPC maturation. Subsequently, A1 astrocytes inhibit microglial phagocytosis of myelin debris via an astrocytic C3-microglial C3aR axis. Polyethylene glycol-CeNP treatment inhibits this pathological process and ultimately promotes remyelination. Such findings enlighten us that astrocytes and microglia should be regarded as a functional unit in future works.

**Supplementary Information:**

The online version contains supplementary material available at 10.1186/s12974-021-02101-6.

## Introduction

Intracerebral hemorrhage (ICH) is a serious neurological crisis that accounts for 20–30% of stroke cases in Asia, with high mortality and disability [1, 2]. White matter accounts for 50–60% of the human brain volume [3, 4]. A previous study demonstrated that 77% of ICH patients had white matter injury (WMI) [5]. ICH commonly occurs around the basal ganglia, which contains abundant white matter fibers (internal capsule). Therefore, ICH can induce severe WMI in patients resulting in hemiplegia, hemianopsia, and sensory deficit.

xIn normal brain tissue, axons are enwrapped by the myelin sheath, which is associated with faster potential propagation and neurological functions [3, 6]. Oligodendrocytes (OLs) are responsible for myelination in the central nervous system (CNS), and individual mature OLs can myelinate up to 60 axons [7]. Previous studies have demonstrated that ICH induced severe oligodendrocyte death and demyelination in white matter [8]. OPCs play a vital role in remyelination as the damaged mature OLs are unable to proliferate and form new myelin sheaths. Although OPCs and immature OLs cannot myelinate axons immediately, OPCs become highly proliferative and then differentiate into mature OLs, which can remyelinate injured axons after demyelinating injuries [9, 10]. The process of OPC proliferation and differentiation is orchestrated by microglia and astrocytes. Microglia are the foremost and earliest immune cells that participate in the pathological process after ICH [2, 11]. In response to microenvironmental cues, microglia may exhibit different polarization states ranging from neurotoxic to protective phenotypes, including M1 and M2 polarized status. Oligodendrocyte proliferation and differentiation can be promoted by M2 anti-inflammatory microglia and impaired by M1 pro-inflammatory microglia [11, 12]. Notably, astrocytes and microglia have a unique bond, and both always respond as one unit when the brain is perturbed [13]. A previous study demonstrated that several CNS diseases resulted in reactive astrocytes, which could be transformed into the A1 phenotype via mediators (IL-1α, C1q, TNF) secreted by activated microglia [14]. The A1 neurotoxic astrocytes can further induce the death of neurons and oligodendrocytes and inhibit OPC maturation. Therefore, there appears to be a strong relationship between microglia-astrocytes crosstalk and remyelination.

Reactive oxygen species (ROS) are excessively produced in perihematomal white matter after ICH, which can oxidize protein, lipids, and nuclear material [15]. Oligodendrocyte-lineage cells (including OPCs and OLs) are rich in lipids and lack essential glutathione and glutathione peroxidase. These specific characteristics make it highly vulnerable to oxidative stress, which ultimately results in WMI [16]. Notably, ROS can induce microglia activation and perturb the kinetics of the M1/M2 shift [17–19]. The scavenging of microglial ROS through genetic abrogation of NADPH oxidase attenuates the pro-inflammatory M1 response and elevates the anti-inflammatory M2 markers [19]. Except for the inflammatory stimuli, ROS have also been implicated in driving astrocyte reactivity [20, 21]. Therefore, ROS scavenging may be a promising way to modulate the activation of microglia and astrocyte and subsequently promote remyelination.

Ceria nanoparticle (CeNP) is known to possess a potent-free radical scavenging activity. The high pseudo-enzyme activity enables 1.0 μg of CeNP to exhibit an equivalent ability to neutralize ROS to 527 U of superoxide dismutase (SOD) [22]**.** Particle miniaturization results in the generation of oxygen vacancies, which leads to the co-existence of Ce3^+^ and Ce4^+^ on the crystalline lattice [18, 23]. As a result, CeNP has SOD mimetic (Ce3^+^) and catalase mimetic (Ce4^+^) together, which enables CeNP to repetitively eradicate ROS [23]. Here, we investigated the putative therapeutic effects of CeNP on WMI via its ROS scavenging capacity, focusing on the role of microglia and astrocytes in remyelination after ICH.

## Method

### Ethics statement

All animal procedures were carried out in accordance with the Guide for the Care and Use of Laboratory Animals of the National Institutes of Health and were approved by the Institutional Animal Care and Use Committee of Zhejiang University. The animals were raised 3 per cage with controlled temperature and humidity and a 12-h light/dark cycle.

### Experimental animals

Adult male C57BL/6 wild type (WT) mice (20–25 g) and mixed-sex C57BL/6 WT mice pups (postnatal within 24 h) were purchased from Slac Laboratory Co., Ltd. (Shanghai, China).

### Administration of PLX3397

The survival of microglia depends on colony-stimulating factor 1 receptor (CSF1R) signaling. Reportedly, dietary treatment with a CSF1R inhibitor PLX3397 effectively depletes microglia in mice without apparent abnormalities in neurological function [24]. For microglia/macrophage depletion, PLX3397 (Selleck chem, Houston, TX) was formulated in AIN-76A standard chow at 290 mg/kg, starting 21 days before ICH surgery until the end of the experiments [24].

### ICH induction

C57BL/6 mice were anesthetized by injecting pentobarbital (40 mg/kg) intraperitoneally and placed in a stereotaxic frame (Stoelting Stereotaxic instrument). A 1-mm burr hole in diameter was made in the skull (0.2 mm anterior to the bregma, 2.2 mm right lateral to the midline). Then, the experimental ICH model was induced by injecting bacterial collagenase VII (0.1 U in 0.5 μL, Sigma, St. Louis, MO) into the right basal ganglia (3.5 mm depth below the skull) using a micro-perfusion pump within 5 min. In case backflows occurred, the needle was not removed for an additional 10 min after the completion of the injection [2, 25]. The burr hole was blocked with bone wax. In the sham group, C57BL/6 mice underwent the same procedures, but 0.5 μL saline was injected instead of collagenase. During the procedure, rectal temperature was maintained at 37.0 ± 0.5 °C using a temperature-regulated heating pad.

### Synthesis of polyethylene glycol (PEG)-CeNPs and administration

As described previously [18], mPEG2000-DSPE 30 mg, 1.1 × 10^− 2^ mmol, was dissolved in 1.0 mL of chloroform. Then it was mixed with 10 mg CeNPs (< 5 nm, Sigma, St. Louis, MO) and stirred for 4 h at room temperature. After removing the chloroform, 10 mL of ultra-pure water was added, and the mixture was dispersed by stirring at 2000 rpm for 2 h, bath sonication for 30 min at 30% amplitude, and full cycle. Finally, the colloidal stabilities of PEG-CeNPs were evaluated by observing their clarity after storage in pure water at 4 °C. The hydrodynamic size distribution and zeta potential of CeNP-PEG in DI water were measured by dynamic light scattering (DLS).

Then, CeNPs (0.5 mg/kg body weight) or an equal volume of phosphate buffer saline (PBS) was intravenously injected at 6 h and 30 h after ICH and then repeated every 3 days

### Dihydroethidium (DHE) injection

DHE was injected intraperitoneally to label superoxide in vivo. At 3 days post ICH, the mice were intraperitoneally injected with DHE (0.01 mg/g body weight). Then, the mice were euthanized 4 h after injection [26]. Afterward, the coronal cryosections were observed using a fluorescence microscope.

### ROS assay

In the brain tissue, ROS production was tested using a ROS assay kit (JianCheng, Nanjing, China) according to the manufacturer’s instructions. Mice were euthanized at 3 days post ICH. The brain tissue samples were lysed in 0.01 mol/L PBS and centrifuged at 4 °C 500 × *g* for 10 min. Then, the supernatant (190 μl) and dichloro-dihydro-fluorescein diacetate (DCFH-DA; 10 μl, 1 mol/L) were mixed in a micro-well at room temperature for 30 min [2]. Then, the protein levels of the different samples were acquired using a detergent-compatible protein assay kit (Bio-Rad, Hercules, CA, USA). Ultimately, the ROS levels were displayed in the form of fluorescence/mg protein. Likewise, the microglia or astrocyte suspensions were incubated with 10 μM DCFH-DA in 96-well plates for 30 min at room temperature. Afterward, these mixtures were measured with a fluorophotometer.

### Transmission electron microscopy (TEM)

As described previously [3], TEM was used to measure myelin thickness in the striatum area. Three mice per group were euthanized at 21 days after ICH and then perfused with 0.9% saline and 2.5% glutaraldehyde. Furthermore, 1-mm^3^ fragments of peri-hematoma tissue were obtained from the right basal ganglia and then processed with glutaraldehyde (2.5%) at 4 °C overnight. Then, the tissues were further handled through a succession of chemical treatment steps (1% osmium tetroxide, and distilled water). Afterward, the samples were embedded in a mixture of propylene oxide and resin (1:1) overnight and were sliced into 60-nm sections and then stained with 4% uranyl acetate and 0.5% lead citrate. The ultrastructure of the myelin was scanned using TEM (Philips Tecnai 10). The electron micrographs were taken at 15 K magnification, and ImageJ software was used to measure the myelin thickness. G-ratios were calculated as the ratio of the inner axonal diameter to the total outer diameter (d/D).

### Flow cytometry

Mice were euthanized and perfused with cold PBS. As described previously [2], the brain tissue was dissociated mechanically and digested in 6 ml of DMEM medium with 0.6 mg/mL collagenase IV (Worthington) for 30 min at 37 °C. Then, the suspension was filtered with a 70-μm cell strainer (BD Biosciences), and the cells were resuspended in 70% Percoll. Then, the cell suspension was separated from the myelin and debris after centrifugation (700 *g*, 4 °C, 40 min) on a 37–70% Percoll gradient. The inflammatory cells were carefully collected from the interface of the gradient and washed with 10 ml of PBS. Single-cell samples were incubated with CD45-percp (1:200, BD PharMingen), CD11b-FITC (1:200, BD PharMingen), CD16/32-PE (1:200, BDPharMingen), and CD206-APC (1:200, BD PharMingen) at 4 °C in the dark for 25 min. CD45^+^CD11b^+^ cells were considered as microglia (CD45^+low^ CD11b^+^) and macrophages (CD45^+high^ CD11b^+^). CD16/32^**+**^ and CD206^**+**^cells were acknowledged as M1 and M2 phenotype microglia, respectively. The subsequent flow cytometric analysis was performed using a FACS flow cytometer C6 (BD Biosciences), and the results were analyzed using FlowJo version 7.6.1.

### Primary microglia and astrocyte culture and treatment

Cortices were isolated from newborn pups (within 24 h) after removing the meninges and blood vessels in Hank’s balanced salt solution and cut into small pieces. The tissue was digested with 2.5% trypsin at 37 °C for 15 min before DMEM was added. Then the single-cell suspension was filtered with a 70-μm cell strainer. After centrifugation and resuspension, mixed glial cells were plated onto poly-d-lysine-coated (PDL) T-25 flasks and cultured with DMEM medium containing 10% fetal bovine serum. These cells were incubated at 37 °C in a humidified 5% CO_2_, 95% air atmosphere. The cell culture medium was changed 24 h after plating, then renewed every 3 days. Then, 7 to 10 days later, microglia were separated from mixed glial cells by orbital shaking at 180 rpm for 1 h. Afterward, the supernatant containing microglia was obtained and re-plated on PDL-coated T-flasks. For the astrocyte culture, the flask with mixed glial cultures was shaken for 3 h at 250 rpm to remove unwanted cells including OPCs, microglia, neurons and fibroblasts. Then the astrocytes were digested with 2.5% trypsin at 37 °C for 5 min and re-plated on T-75 flasks [27, 28].

Microglia were incubated with H_2_O_2_ 100 μM for 24 h. Astrocytes were incubated with H_2_O_2_ 100 μM for 6 h. In the treatment group, 0.32 μg/mL of PEG-CeNPs was incubated with microglia or astrocytes for 4 h and then washed twice with PBS before H_2_O_2_ treatment [18].

### Nissl staining

Coronal cryosections were stained with Nissl staining solution (Beyotime, C0117) for 30 min at 37 °C. Then, the cryosections were washed using 95% ethyl alcohol and observed using an optical microscope. A large cell body, with abundant cytoplasm and substantially significant levels of Nissl body, represents a normal neuron. Cells with karyopyknosis or blurred Nissl bodies represented damaged ones.

### Immunofluorescence staining and image analysis

The mice were euthanized under deep pentobarbital anesthesia at 3, 7, and 21 days after ICH, and then perfused with 0.1 mol/L PBS and 4% paraformaldehyde (PFA). The coronal cryosections and primary cultured cells (microglia and astrocytes) were preprocessed with 10% donkey serum and 0.3% Triton X-100, and then incubated at 4 °C with different antibodies overnight including MBP antibody (1:200, Santa Cruz, sc-66064), degraded myelin basic protein, and dMBP antibody (1:400, Millipore, AB5864)

Anti-CD16/32 antibody (1:100, Abcam ab25235), anti-CD206 antibody (1:500, Abcam ab64693), Iba-1 (1:500, Abcam ab5076), anti-NF-κB p65 antibody (1:500, CST #6956), anti-olig2 antibody (1: 250, Millipore AB9610), anti-CC1 antibody (1: 500, Millipore OP80), anti-GFAP (Glial Fibrillary Acidic Protein) antibody (1:500, CST #34001S), anti-Complement C3 antibody (1:400, Invitrogen PA5-21349), and anti-C3aR antibody (1:100, Hycult Biotech #HM1123). Afterward, the cryosections or cultured cells were incubated with the secondary antibodies. Then, a fluorescence microscope (Olympus, Tokyo, Japan) was used to capture the images.

For MBP and dMBP staining, the areas of white matter lesion were assessed for each mouse brain. The areas of the MBP lesion or dMBP positive region were measured using ImageJ software. Likewise, positive staining cells were electronically counted using ImageJ software. Afterward, these data were calculated and analyzed.

### Phagocytosis assay

Microglia were preprocessed with 10 μg/ml of C3 (Millipore; catalog no. #204885) or A1 astrocyte conditioned medium (CM) for 24 h. For the preparation of A1 astrocyte CM, A1 astrocytes were maintained in basal medium without serum or growth factors for 24 h. Collected conditioned medium were centrifuged (2000 *g*, 10 min) to remove debris and then filtered through a 0.22-μm syringe filter. The A1 astrocyte CM was stored at − 80 °C before use. In the C3aR antagonist (C3aRA, SB290157, MCE HY-101502A) + A1 astrocyte CM group, 10 μM of C3aRA was incubated with microglia for 1 h before the A1 astrocyte CM treatment. Then, aqueous green fluorescent beads (1 μm diameter, Sigma, #L1030) were added into the medium for 3 h. The final concentration (v/v) of beads and fetal bovine serum in the medium were 0.01% and 0.05%, respectively [29].

### Quantitative real-time polymerase chain reaction (RT-PCR)

Total ribonucleic acid (RNA) was isolated from the mice brain tissue or primary cultured astrocyte using TRIzol reagent (Sigma-Aldrich, St. Louis, MO, USA), following the manufacturer’s protocol. Then, cDNA synthesis was performed using a PrimeScriptTM RT reagent kit (Takara Bio Inc, Shiga, Japan). Afterward, cDNAs were amplified using SYBR® Premix Ex Taq™ (Takara Bio Inc, Shiga, Japan) on a 7300 Plus Read-Time PCR System (Thermo Fisher Scientific). The PCR reaction was performed as follows. The cycling conditions began with an initial DNA denaturation step at 95 °C for 20 s, followed by 40 cycles at 94 °C for 15 s, 56 °C for 30 s, and 72 °C for 25 s. The cycle threshold values were collected and calculated with the 2^−ΔΔCT^ method. Finally, mRNA expression was shown as fold changes versus sham controls. The target gene primers designed for quantitative RT-PCR are listed in Table 1.

**Table 1:** RT-PCR primer sequences

ID		Primer sequence
*iNOS*	FWD	CAAGCACCTTGGAAGAGGAG
	REV	AAGGCCAAACACAGCATACC
*CD16*	FWD	TTTGGACACCCAGATGTTTCAG
	REV	GTCTTCCTTGAGCACCTGGATC
*IFN-γ*	FWD	CAGCAACAGCAAGGCGAAAAAGG
	REV	TTTCCGCTTCCTGAGGCTGGAT
*CD206*	FWD	CAAGGAAGGTTGGCATTTGT
	REV	CCTTTCAGTCCTTTGCAAGC
*YM1/2*	FWD	CAGGGTAATGAGTGGGTTGG
	REV	CACGGCACCTCCTAAATTGT
*TGF-β*	FWD	TGCGCTTGCAGAGATTAAAA
	REV	CGTCAAAAGACAGCCACTCA
*C3*	FWD	CCAGCTCCCCATTAGCTCTG
	REV	GCACTTGCCTCTTTAGGAAGTC
*Serping1*	FWD	ACAGCCCCCTCTGAATTCTT
	REV	GGATGCTCTCCAAGTTGCTC
*Amigo2*	FWD	GAGGCGACCATAATGTCGTT
	REV	GCATCCAACAGTCCGATTCT
*GAPDH*	FWD	AAGAGGGATGCTGCCCTTAC
	REV	TACGGCCAAATCCGTTCACA

### Western blotting

Proteins were isolated from the mice brain tissue or cultured cells. Nuclear and cytoplasmic protein was separated using a Nuclear and Cytoplasmic Protein Extraction Kit (Beyotime, P0028). The cells or peri-hematoma tissues (basal ganglia) were homogenized in radio-immunoprecipitation assay lysis buffer (Beyotime). Then, the protein samples were separated by 10% or 12% SDS-PAGE and transferred onto polyvinylidene fluoride (PVDF) membranes (Millipore). Next, the polyvinylidene fluoride (PVDF) membranes were blocked with 5% bovine serum albumin for 1 h and incubated with the primary antibodies overnight, including anti-phosphorylated NF-κB p65 antibody (1:500, Santa Cruz, sc-136548), anti-NF-κB p65 antibody (1:1000, CST #6956), anti-IκB-α antibody (1:500, Santa Cruz, sc-1643), MBP antibody (1:500, Santa Cruz, sc-66064), Histone H3 (1:1000, CST #9715) and β-actin (1:5000, Abcam, ab8226). After that, the PVDF membranes were disposed of with the relevant secondary antibodies (1:5000) for 1 h at room temperature. The signals of the protein bands were detected using a Chemidoc detection system and quantified using Quantity One software (Bio-Rad).

### Behavioral tests

#### Accelerated rotarod test

An accelerated rotarod test was performed using Rotarod Treadmills (BW-ZH600, Shanghai Bio-will Co., Ltd.) to test the motor coordination and limb strength of the mice. As described previously [25], the mice were placed on a six-lane accelerating rotarod (acceleration from 4 to 40 rpm within 5 min, increasing 4 rpm every 30 s until reaching the final speed at 300 s). The time during which the animals stayed on the rotarod was recorded. Each animal underwent this test 3 times a day. Data were expressed as the mean values from the 3 trials.

#### Adhesive removal test

As described previously [2, 3], the adhesive removal test was performed by an independent researcher to detect the tactile responses of the mice. Before the actual test, mice had been trained for 3 days to familiarize them with the test. Then, adhesive tape (3 mm × 3 mm) was pasted on the left forepaw (affected side). The time to contact and remove the adhesive tape was measured (a maximum observation time 120 s).

### Measurement of brain water content

As described previously, the brain water content was measured using wet-dry method [2]; mice were euthanized under deep pentobarbital anesthesia at 3 days after ICH. After removal of cerebellum and brain stem, the ipsilateral hemisphere was immediately weighed on an analytical balance (wet weight), and then dried at 100 °C for 24 h in the Electro-Thermostatic Blast Oven. The dry weight of these brain tissues was acquired by re-weighting. Ultimately, brain water content was calculated using the following formula: [(wet weight-dry weight)/wet weight] × 100%.

### EdU injection

In order to label proliferating cells, mice were intraperitoneally injected with a thymidine analog (5-ethynyl-2′-deoxyuridine, EdU) at a dose of 50 mg/kg body weight. Mice were injected with EdU (Beyotime, Shanghai, China, C0075L) twice a day (with an interval of at least 8 h) at 3 days after ICH for 4 consecutive days. Then EdU can be incorporated into cellular DNA and the subsequent reaction of EdU with a fluorescent azide in a copper-catalyzed [3 + 2] cycloaddition (Click reaction) [3, 30].

### Study design

Animals were sacrificed as little as possible through rational experimental design, a total of 242 mice (including 30 neonatal mice) were used for this study at last. The detailed information is shown in Figure S1. The data collected was processed randomly and appropriately blocked. In addition, researchers were partly blind to group assignment and outcome assignment.

#### Experiment 1

In order to evaluate the effectiveness of PEG-CeNP, 12 mice were randomly (using random number table) divided into 2 groups: ICH 3d + vehicle (PBS with PEG) *n* = 6, ICH 3 days + PEG-CeNP, *n* = 6. Then, these animals were applied to Nissl staining. To observe relevant ROS levels, 12 mice were undergone DHE injection (ICH 3d + vehicle *n* = 6, ICH 3d + PEG-CeNP, *n* = 6), another 18 mice (Sham *n* = 6, ICH 3 days + vehicle *n* = 6, ICH 3 days + PEG-CeNP, *n* = 6) were used the quantify the ROS production by using a ROS assay kit. In total, 18 mice (Sham *n* = 6, ICH 3 days + vehicle *n* = 6, ICH 3 days + PEG-CeNP, *n* = 6) were euthanized and used for brain water content test. Moreover, 9 mice (Sham *n* = 3, ICH 21 days + vehicle *n* = 3, ICH 21 days + PEG-CeNP, *n* = 3) were euthanized for TEM analysis to evaluate white matter injury, and 12 mice (ICH 21d + vehicle, *n* = 6, ICH 21 days + PEG-CeNP, *n* = 6) were undergone MBP and dMBP immunofluorescence staining. These aforementioned animals (TEM and MBP staining) were also applied to behavioral test on days 0, 1, 3, 7, 14 and 21 days, *n* = 9.

#### Experiment 2

In vivo and in vitro studies were used to investigate whether PEG-CeNP treatment modulated microglial polarization via inhibiting ROS-induced NF-κB p65 translocation. 30 mice were randomly divided into 5 groups: Sham group *n* = 6, ICH 3 days *n* = 6, ICH 3d + PEG-CeNP *n* = 6, ICH 7 days *n* = 6, ICH 21 days *n* = 6. Then, these animals were euthanized for PCR to detect the expression of M1/M2 microglia marker. In addition, 12 mice (ICH 3 days + vehicle *n* = 6, ICH 3 days + PEG-CeNP *n* = 6) were undergone immunofluorescence staining (CD1632/Iba1, CD206/Iba1). Moreover, 24 mice (ICH 3 days + vehicle *n* = 12, ICH 3 days + PEG-CeNP *n* = 12) were applied for flow cytometry and western blotting. The in vitro experiments were performed to further investigate the effects of PEG-CeNP on microglia.

#### Experiment 3

To investigate PEG-CeNP promoted OPC differentiation/maturation partly in a microglia-dependent manner, PLX3397 was used for microglia depletion in vivo. In total, 20 mice (Sham *n* = 5, Sham + PLX3397 *n* = 5, ICH 7 days + vehicle *n* = 5, ICH 7 days +PLX3397 *n* = 5) were applied to flow cytometry and Iba1 immunostaining. Moreover, 24 mice (ICH 7 days + vehicle *n* = 6, ICH 7 days + PEG-CeNP *n* = 6, ICH 7 days + vehicle + PLX3397 *n* = 6, ICH 7 days + PEG-CeNP + PLX3397 *n* = 6) were undergone olig2/CC1 immunostaining. In addition, 24 mice (ICH 7 days + vehicle *n* = 6, ICH 7 days + PEG-CeNP *n* = 6, ICH 7 days + vehicle + PLX3397 *n* = 6, ICH 7 days + PEG-CeNP + PLX3397 *n* = 6) were undergone EdU injection and the following EdU/CC1 immunostaining.

#### Experiment 4

In vivo and in vitro studies were used to investigate whether PEG-CeNP treatment protected against A1 astrocyte alteration after ICH. A total of 30 mice were randomly divided into 4 groups: Sham group *n* = 6, ICH 3 days *n* = 6, ICH 7 days *n* = 6, ICH 7 days + PEG-CeNP *n* = 6, ICH 21d *n* = 6. Then, these animals were euthanized for PCR to detect the expression of A1 astrocyte marker. Then, 12 mice (ICH 7 days + vehicle *n* = 6, ICH 7 days + PEG-CeNP *n* = 6) were undergone C3/GFAP immunofluorescence staining. In vitro, NF-κB p65/GFAP immunostaining and western blotting were performed to investigate whether PEG-CeNP treatment inhibited A1 astrocyte via inhibiting ROS-induced NF-κB p65 translocation.

#### Experiment 5

To investigate whether A1 astrocytes inhibited microglial phagocytosis of myelin debris via an astrocytic C3-microglial C3aR axis, Phagocytosis assay was performed by using fluorescent beads in vivo.

### Statistical analysis

Results are shown as the mean ± standard error of the mean (SEM). Student’s *t* test was used to compare 2 groups for continuous variables with normal distributions. The Mann-Whitney *U* rank-sum test was used for continuous variables with non-normal distributions. For multiple groups, a one-way analysis of variance (ANOVA) with Tukey’s post hoc analysis was used. A two-way ANOVA with Tukey’s multiple comparisons test was used to assess the significance of behavioral tests in different groups at various time points. Data analysis was conducted by investigators blinded to experimental groups. Sample sizes for the animal studies were determined based on pilot studies or the current literature. Statistical Package for the Social Sciences (version 22.0) and Prism (version 8.0) were used for the statistical analysis. A *p* value of < 0.05 indicates statistical significance.

## Results

### ICH-induced severe white and gray matter injury

**Fig. 1:**
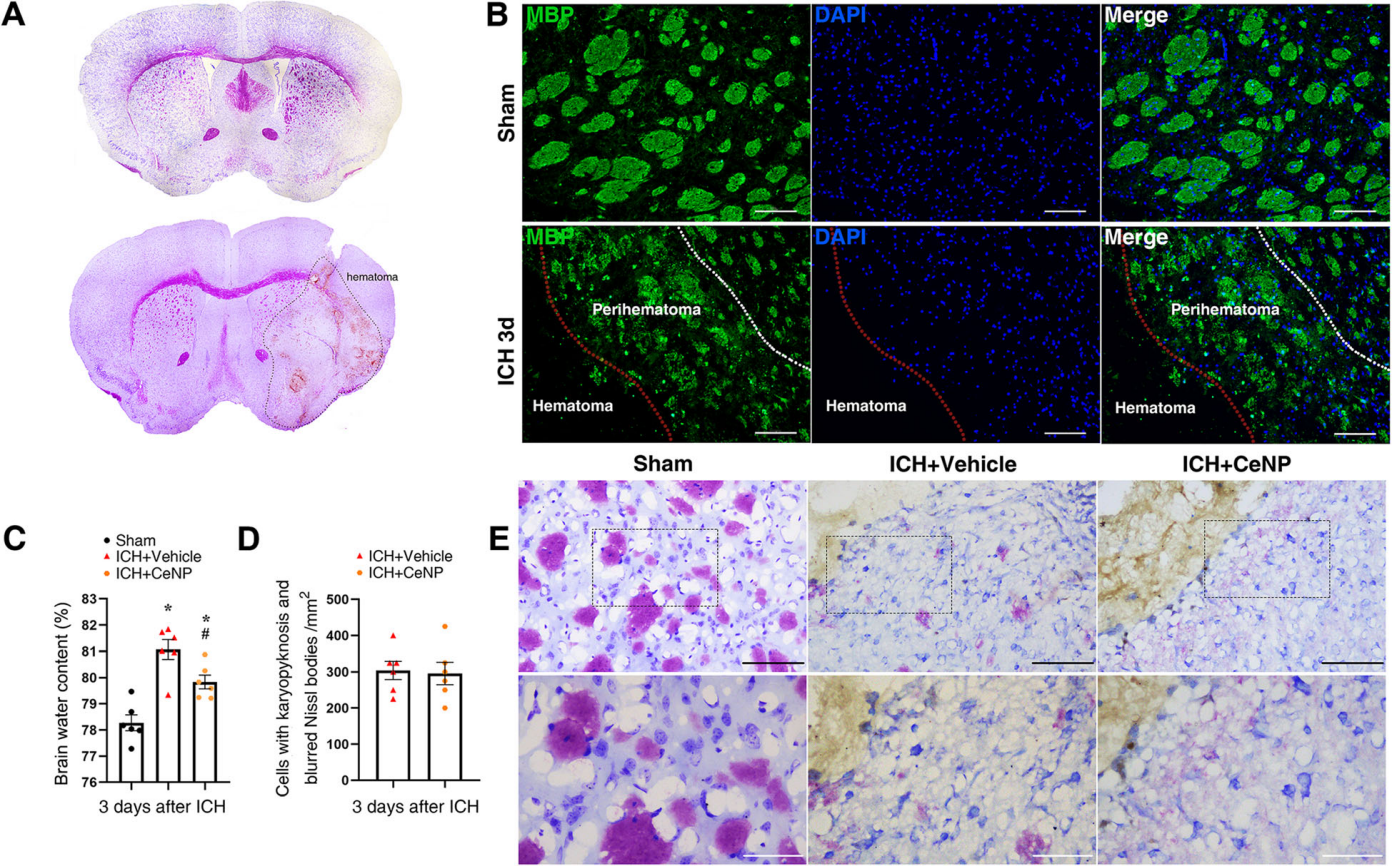
ICH-induced white matter injury. **a** Nissl’s staining showed severe gray and white matter injury after experimental ICH in mice. **b** Representative images of MBP immunostaining at 3 days post ICH. Lens: × 200; Scale bar: 100 μm. **c** Measurement of brain water content at 3 days post ICH. * *p* < 0.05 versus Sham, # *p* < 0.05 versus ICH + vehicle. **d** Quantification of neurons with karyopyknosis and blurred Nissl bodies, *n* = 6/group. **e** Nissl’s staining of injured neurons. Lens: × 200, × 400; Scale bar: 100 μm (black), 50 μm (white)

Nissl’s staining showed that the injection of collagenase VII caused the striatum hemorrhage, which induced severe white and gray matter injury (Fig. 1a). The MBP immunofluorescence staining also showed significant structural damage in the myelin sheaths at 3 days post ICH (Fig. 1b).

CeNPs were modified with a PEG coating to have better biocompatibility and avoid interparticle agglomeration. As shown in Figure S2, PEG-CeNPs showed better colloidal stability than normal CeNPs after storage in pure water at 4 °C. PEG-CeNP treatment significantly attenuated brain edema at 3 days after ICH (Fig. 1c, mean ± SEM: 79.83 ± 0.26% (ICH + CeNP) versus 81.06 ± 0.38% (ICH + vehicle), *p* < 0.05). However, PEG-CeNP treatment failed to decrease the number of injured neurons (Fig. 1d, e, *p* > 0.05).

### PEG-CeNP treatment reduced ROS accumulation

**Fig. 2:**
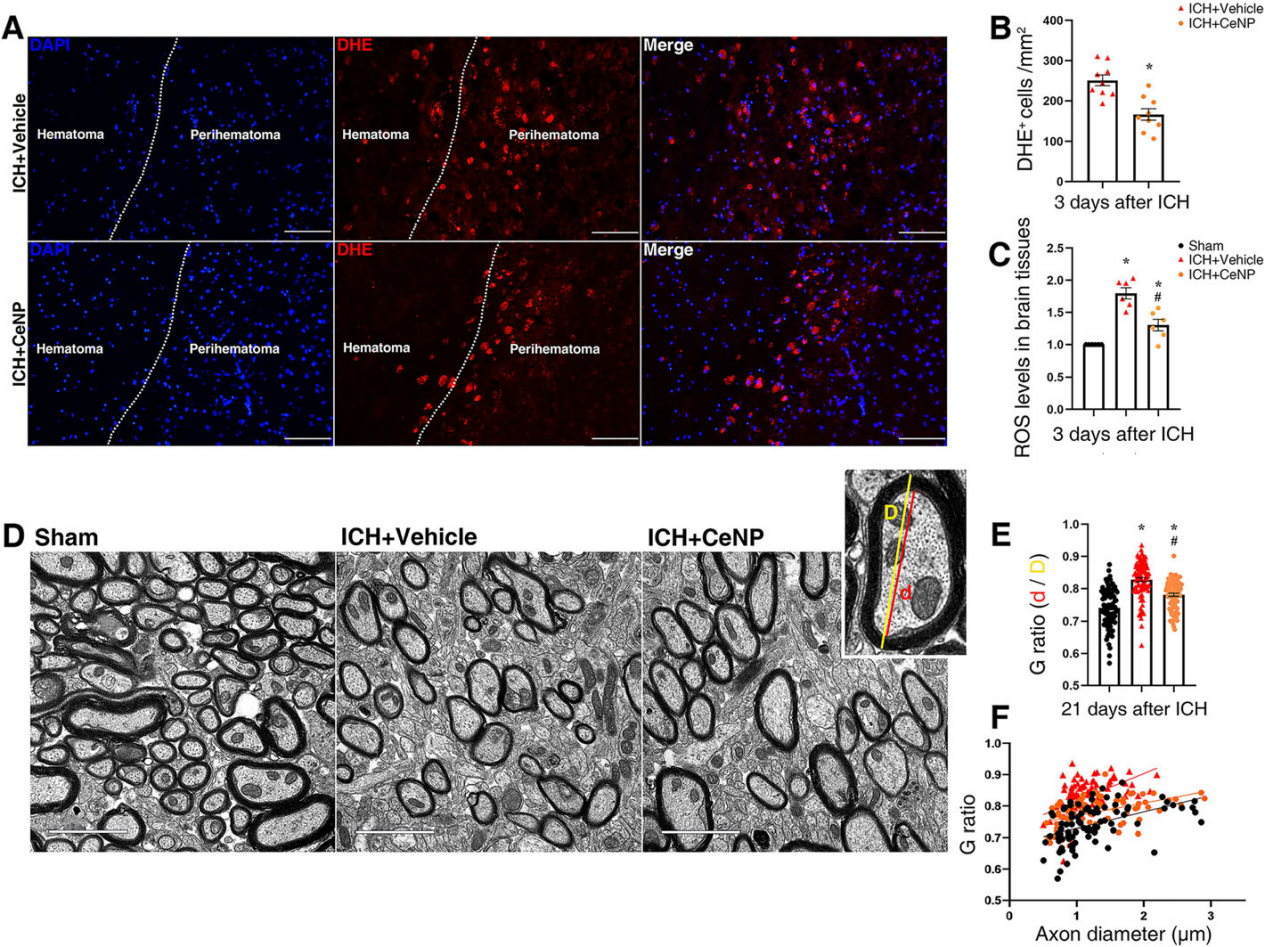
PEG-CeNP treatment reduced ROS accumulation and improved anatomical integrity of myelinated fibers after ICH. **a** Representative images in peri-hematoma site stained with the superoxide-sensitive dye DHE at 3 days post ICH. Lens: × 200; Scale bar: 100 μm. **b** Quantification of DHE+DAPI+ cells, *n* = 3/group, * *p* < 0.01 versus ICH + vehicle. **c** ROS levels in brain tissues at 3 days post ICH, *n* = 6/group, * *p* < 0.01 versus Sham, # *p* < 0.01 versus ICH + vehicle. **d** Representative electron micrographs showed myelin sheaths in striatum at 21 days post ICH. Lens: × 15,000; Scale bar: 2 μm. **e** Histograms show quantifications of the G-ratio at 21 days post ICH, *n* = 3/group, * *p* < 0.01 versus Sham, # *p* < 0.01 versus ICH + vehicle. **f** Scatter plots of G-ratio against axon diameter

To investigate the free radical scavenging activity of PEG-CeNP in vivo, the superoxide-sensitive dye DHE was injected intraperitoneally at 3 days post ICH (Fig. 2a). Quantification of DHE-positive cells indicated that PEG-CeNP treatment significantly reduced ROS accumulation (Fig. 2b, mean ± SEM: 250.9 ± 13.6/mm^2^ (ICH + CeNP) versus 165.9 ± 14.2/mm^2^ (ICH + vehicle), *p* < 0.01). Likewise, the data of ROS assay in the brain tissues also supported the results mentioned above (Fig. 2c, ICH + CeNP versus ICH + vehicle, *p* < 0.01).

### PEG-CeNP treatment promoted remyelination and long-term neurological function

**Fig. 3:**
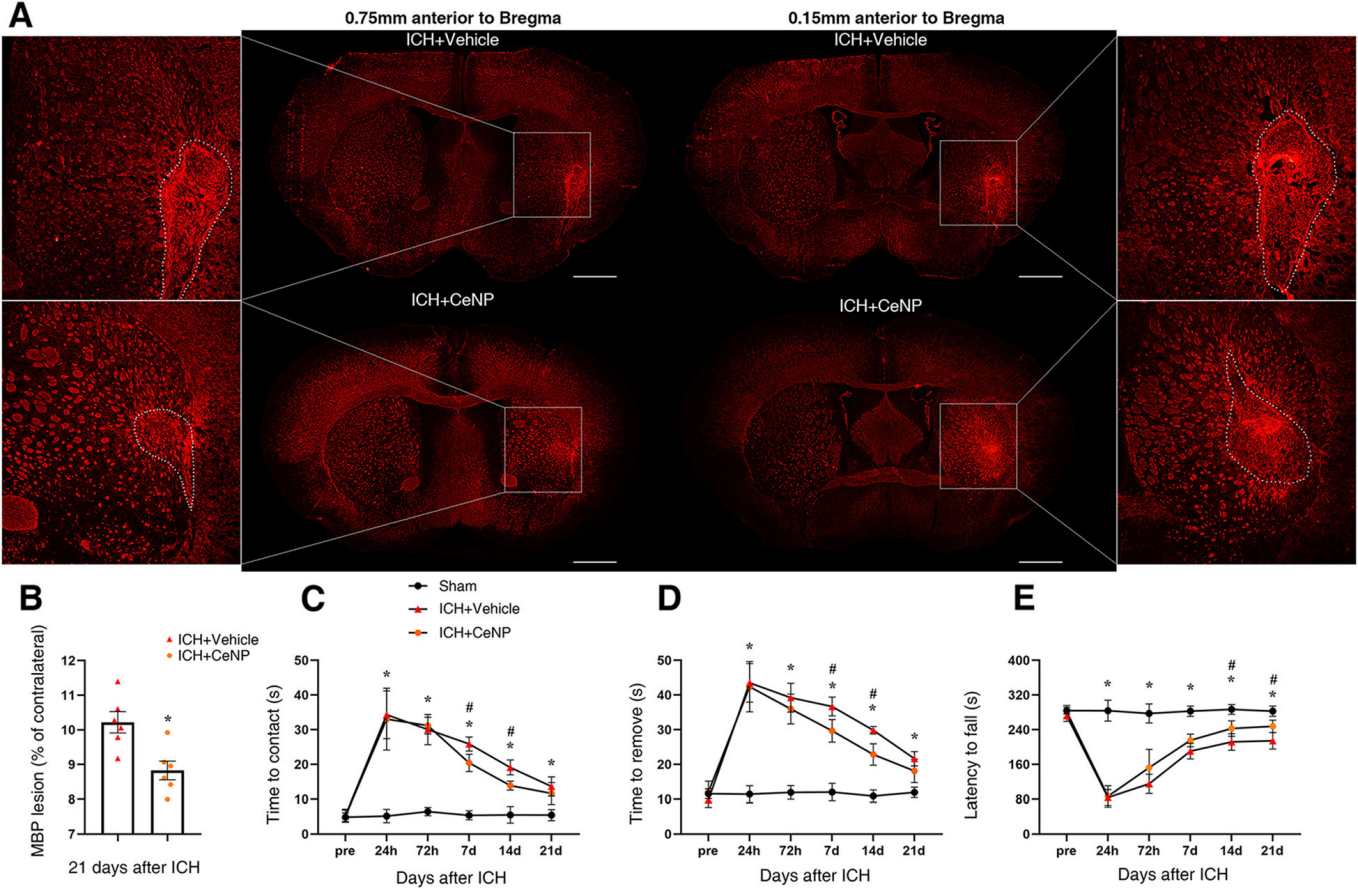
PEG-CeNP treatment reduced white matter injury and improved long-term neurological functions after ICH. **a** Representative images of MBP immunostaining in brain sections at 21 days post ICH. Lens: × 50; Scale bar: 500 μm. **b** % of MBP lesion area compared to the MBP+ area of contralateral hemisphere. *n* = 6/group, * *p* < 0.05 versus ICH + vehicle. **c** Adhesive removal test, time to contact. **d** Adhesive removal test, time to remove. **e** Accelerated rotarod test, latency to fall. *n* = 9/group, * *p* < 0.01: ICH+ CeNP and ICH + vehicle versus Sham. # *p* < 0.05: ICH+ CeNP versus ICH + vehicle

To investigate whether PEG-CeNP treatment can promote remyelination, TEM analysis was performed to measure the thickness of the myelin sheaths at the peri-hematoma site in the striatum. Representative electron micrographs showed the myelin sheaths in the peri-hematoma region at 21 days post ICH (Fig. 2d). The results showed that the myelin sheaths in ICH mice displayed a higher G-ratio than those in the sham group (Fig. 2e, f, *p* < 0.01 versus sham, average G-ratio: sham 0.74 ± 0.0063, ICH + vehicle 0.82 ± 0.006, ICH + CeNP 0.78 ± 0.005), indicating a reduction in the myelin thickness. By contrast, mice in the ICH+ CeNP group showed a lower G-ratio compared to the ICH mice (Fig. 2e, f, *p* < 0.01 versus ICH + vehicle). Representative imaging of the MBP immunostaining displayed the area of the white matter lesion at 21 days post ICH (Fig. 3a). PEG-CeNP treatment significantly decreased the white matter lesion compared to ICH + vehicle (Fig. 3b, *p* < 0.01 versus ICH + vehicle). Namely, PEG-CeNP treatment promoted remyelination and improved anatomical integrity of the myelinated fibers.

Adhesive removal test was applied 1, 3, 7, 14, and 21 days after ICH. PEG-CeNP-treated mice and vehicle-treated mice displayed a significant reduction in contact and removal time compared to the sham group (Fig. 3c, d, *p* < 0.01 versus sham). This indicated that ICH caused significant sensorimotor deficit. PEG-CeNP treatment significantly alleviated such sensorimotor deficit at 7 and 14 days post ICH (Fig. 3c, d, *p* < 0.05 versus ICH + vehicle). The accelerated rotarod test showed that the motor coordination and limb strength of mice were significantly decreased after ICH induction in both PEG-CeNP-treated mice and vehicle-treated mice (Fig. 3e, *p* < 0.01 versus sham). Long-term motor coordination and limb strength in the rotarod test at 14 and 21 days post ICH were significantly improved in PEG-CeNP-treated mice (Fig. 3e, *p* < 0.01 versus ICH + vehicle).

### PEG-CeNP treatment-induced microglia skewed polarization

**Fig. 4:**
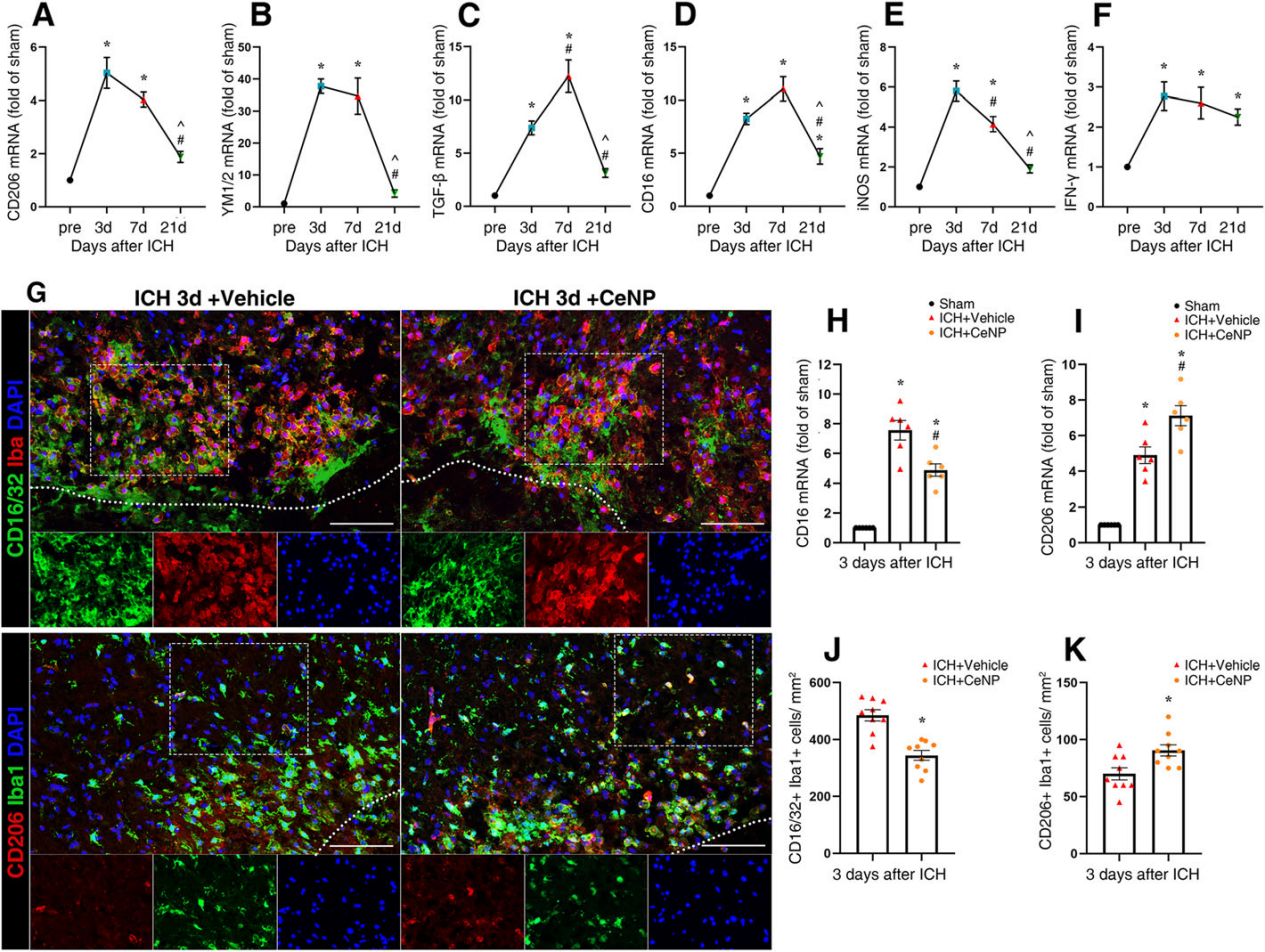
PEG-CeNP treatment-induced microglia skewed polarization after ICH. Representative RT-qPCR analysis of M2 (**a**–**c**) and M1 (**d**–**f**) microglia markers expression. *n* = 6/group, * *p* < 0.01 versus Sham. # *p* < 0.01 versus ICH 3d. ^ *p* < 0.01 versus ICH 7d. **g** Representative images of CD1632/Iba1 and CD206/Iba1 double immunostaining at 3 days post ICH. Lens: × 200; Scale bar: 100 μm. **h** CD16 mRNA expression in PEG-CeNP-treated mice at 3 days post ICH. *n* = 6/group, * *p* < 0.01 versus Sham, # *p* < 0.01 versus ICH + vehicle. **i** CD206 mRNA expression in PEG-CeNP-treated mice at 3 days post ICH. *n* = 6/group, * *p* < 0.01 versus Sham, # *p* < 0.05 versus ICH + vehicle. **j** Quantification of CD16/32^**+**^Iba1^**+**^ cells, *n* = 6/group, *p < 0.01 versus ICH + vehicle. **k** Quantification of CD206^**+**^Iba1^**+**^ cells, *n* = 6/group, * *p* < 0.05 versus ICH + vehicle

To investigate the polarization dynamics of microglia after ICH, RT-PCR was used to test the mRNA expression of M1 and M2 microglia markers in the brain tissue 3, 7, and 21 days after ICH. As shown in Fig. 4a–c, the mRNA expression of these classic M2 markers (CD206, YM1/2, TGF-β) peaked at 3 or 7 days after ICH (*p* < 0.01). The difference became less obvious at 21 days post ICH (Fig. 4a–c, *p* > 0.05 versus sham). The mRNA expression of these classic M1 markers, CD16 and iNOS, peaked at 3 or 7 days after ICH, except for IFN-γ (Fig. 4d–f, *p* < 0.01). However, the mRNA expression of IFN-γ increased after ICH induction (*p* < 0.01 versus sham). The difference between these three groups (ICH 3, 7, and 21 days) was not statistically significant (*p* > 0.05).

In light of the results mentioned above, day 3 was chosen as the specific time point to explore the effects of PEG-CeNP in ICH mice. PEG-CeNP treatment significantly inhibited CD16 mRNA expression compared to ICH + vehicle (Fig. 4h, *p* < 0.01 versus ICH + vehicle). In addition, CD206 mRNA expression was slightly increased in PEG-CeNP-treated mice (Fig. 4i, *p* < 0.05 versus ICH + vehicle).

Since the mRNA expression in brain tissue could not specifically reflect the changes in microglia, immunofluorescence staining was conducted to identify the expression of M1 and M2 markers in microglia after ICH (Fig. 4g). The immunofluorescence staining of CD206 and CD16/32 at 3, 7, and 21 days post ICH showed good consistency with mRNA expression time curves (Fig. 4g ICH 3 days, Figure S3 ICH 7 days, Figure S4 ICH 21d). The number of CD16/32^**+**^ Iba1^**+**^ cells were significantly decreased in PEG-CeNP-treated mice (Fig. 4j, mean ± SEM: 485 ± 19.57 (ICH + vehicle) versus 344 ± 17.16 (ICH + CeNP), *p* < 0.01). CD206^**+**^ Iba1^**+**^ cells were moderately increased in ICH mice with PEG-CeNP treatment (Fig. 4k, mean ± SEM: 70 ± 5.33 (ICH + vehicle) versus 90 ± 4.89 (ICH + CeNP), *p* < 0.05).

### PEG-CeNP treatment modulated microglial polarization via inhibiting ROS-induced NF-κB p65 translocation

**Fig. 5:**
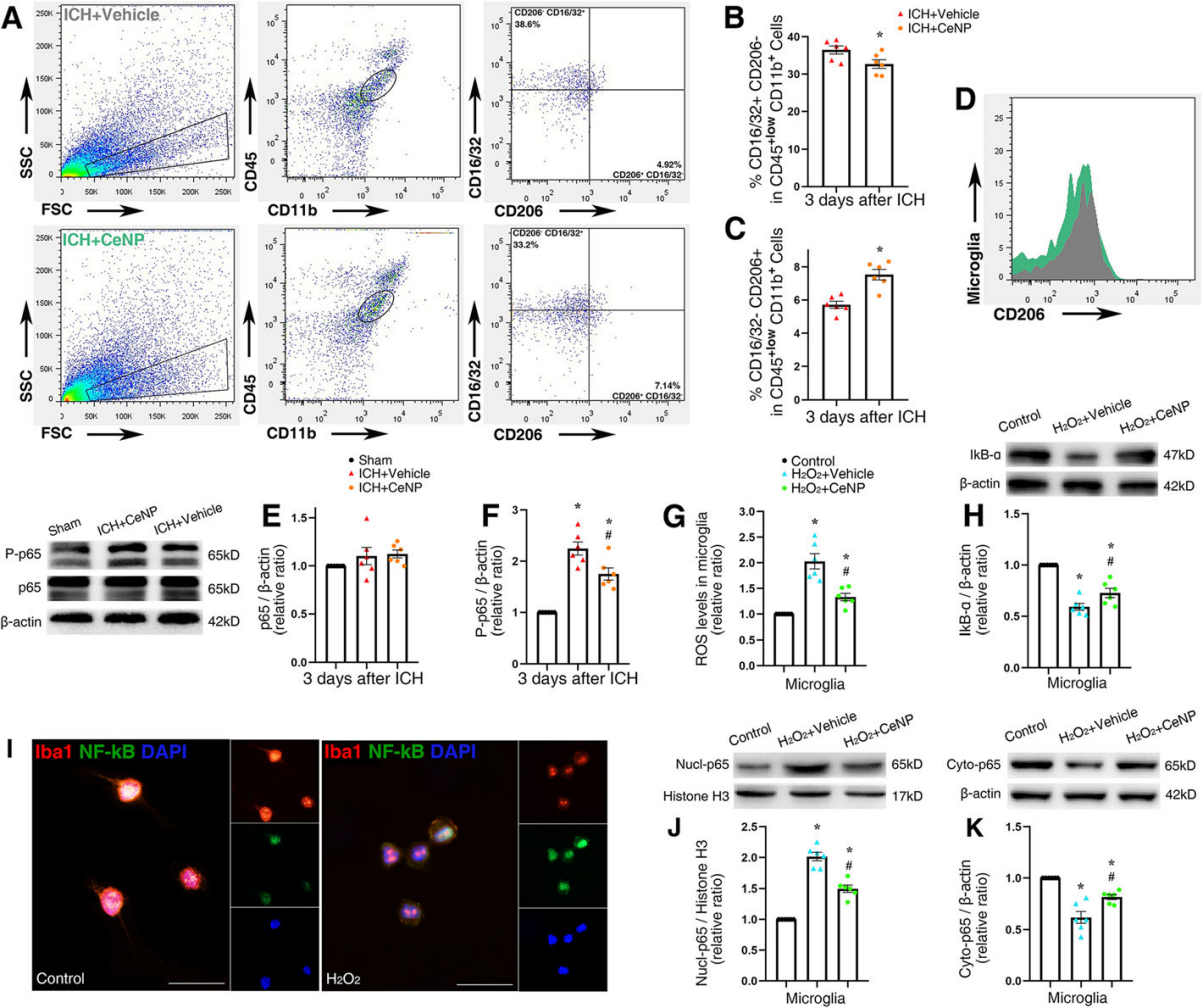
PEG-CeNP treatment modulated microglial polarization via inhibiting ROS-induced NF-κB p65 translocation. **a** Flow cytometric analysis of microglia isolated from the ipsilateral hemisphere at 3 days post ICH. Representative gating strategy for microglia (CD45^low+^CD11b^+^), macrophages (CD45^high+^CD11b^+^), M1 microglia (CD16/32^+^CD206^−^,CD45^low+^CD11b^+^), and M2 microglia (CD16/32^−^CD206^+^,CD45^low+^CD11b^+^). **b** Quantification of CD16/32^**+**^CD206^−^ microglia, *n* = 6/group, *p < 0.01 versus ICH + vehicle. **c** Quantification of CD16/32^−^CD206^**+**^ microglia, *n* = 6/group, * *p* < 0.05 versus ICH + vehicle. **d** Mean fluorescence intensity of CD206 in ICH + vehicle (gray) and ICH+ CeNP (green) group. **e, f** Representative Western blot bands showed the expression of NF-κB p65 and phosphorylated NF-κB p65 (P-p65) in brain tissues at 3 days after ICH, *n* = 6/group, * *p* < 0.05 versus Sham, # *p* < 0.05 versus ICH + vehicle. **g** ROS levels in cultured microglia, *n* = 6/group, * *p* < 0.05 versus Control, # *p* < 0.05 versus H_2_O_2_ + vehicle. **h** Representative Western blot bands showed the expression of IκB-α in cultured microglia, *n* = 6/group, * *p* < 0.05 versus Control, # *p* < 0.05 versus H_2_O_2_ + vehicle. **i** Representative images of NF-κB p65 and Iba1 double immunostaining in cultured microglia. Lens: × 400; Scale bar: 25 μm. **j**, **k** Representative Western blot bands showed the expression of nuclear and cytoplasm NF-κB p65 in cultured microglia, *n* = 6/group, * *p* < 0.05 versus Control, # *p* < 0.05 versus H_2_O_2_ + vehicle

Flow cytometric analysis was performed to quantify further the proportion of M1 and M2 microglia in the brain tissue. The representative gating strategy for microglia (CD45^**low+**^ CD11b^**+**^), macrophages (CD45^**high+**^ CD11b^**+**^), M1 microglia (CD16/32^**+**^CD206^−^, CD45^**low+**^CD11b^**+**^), and M2 microglia (CD16/32^-^CD206^**+**^, CD45^**low+**^CD11b^**+**^) is shown in Fig. 5a. The quantity of CD16/32^**+**^CD206^−^ microglia were decreased after PEG-CeNP treatment compared to ICH + vehicle (Fig. 5b, *p* < 0.05). PEG-CeNP treatment also increased the number of CD16/32^**-**^CD206^+^ microglia (Fig. 5c, *p* < 0.05 versus ICH + vehicle). The mean fluorescence intensity of CD206 was also in accordance with the results mentioned previously (Fig. 5d).

Microglial phenotypes are tightly controlled by ROS levels. As a traditional transcription factor, NF-κB plays a crucial role in microglia activation. ROS can induce NF-κB p65 activation and translocation, and then regulate microglia polarization [11, 31]. Hence, we detected the protein expression of phosphorylated NF-κB p65 (P-p65) and p65 in mice brain tissue after ICH induction. The western blot results indicated that ICH significantly increased P-p65 expression (Fig. 5e, f, *p* < 0.05 versus sham), and PEG-CeNP treatment decreased P-p65 expression (Fig. 5e, f, *p* < 0.05 versus ICH + vehicle).

In cultured microglia, H_2_O_2_ was used to induce microglia activation and ROS accumulation. As shown in Fig. 5g, PEG-CeNP displayed remarkable free radical scavenging activity in vitro. ROS levels in microglia treated with H_2_O_2_ were significantly reduced after PEG-CeNP treatment (*p* < 0.05 versus H_2_O_2_ + vehicle). Representative images of NF-κB p65 and Iba1 double immunostaining in cultured microglia indicated that H_2_O_2_ treatment resulted in obvious translocation of NF-κB p65 from the cytoplasm to the nucleus (Fig. 5i). Also, the western blot analysis revealed that ROS accumulation induced the downregulation of IκB-α (inhibitor of NF-κB) and cytoplasmic p65 in microglia (Fig. 5h, k, *p* < 0.05 versus control). In contrast, nuclear NF-κB p65 was significantly increased in microglia after H_2_O_2_ stimulation (Fig. 5j, *p* < 0.05 versus control). PEG-CeNP treatment inhibited IκB-α degradation and the translocation of NF-κB p65 from the cytoplasm to the nucleus (Fig. 5h, j, k, *p* < 0.05 versus H_2_O_2_ + vehicle).

### PEG-CeNP promoted OPCs differentiation, partly in a microglia-dependent manner

**Fig. 6:**
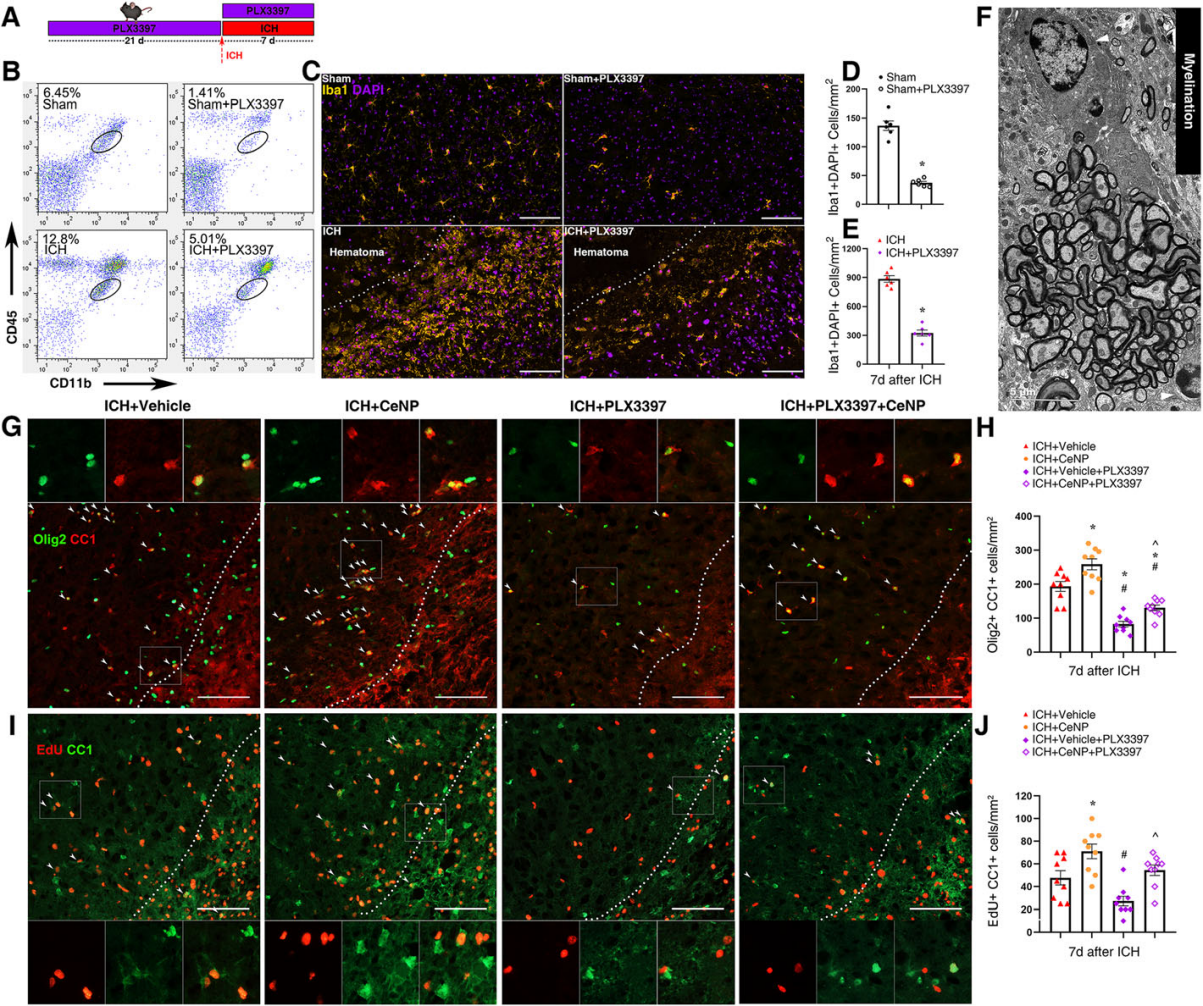
PEG-CeNP promoted OPCs differentiation/maturation partly in a microglia-dependent manner. **a** PLX3397 administration. **b** Representative flow cytometric dot-plot showed the percentage of microglia after PLX3397 administration in different groups. **c** Representative fluorescent images showed the expression of Iba1^**+**^ Cells after PLX3397 administration in striatum. Lens: × 200; Scale bar: 100 μm. **d** Quantification of Iba1^**+**^ Cells in Sham and Sham+PLX3397 group, *n* = 3/group, * *p* < 0.01 versus Sham. **e** Quantification of Iba1^**+**^ Cells in ICH and ICH + PLX3397 group, *n* = 3/group, * *p* < 0.01 versus ICH. **f** Representative TEM image of myelination showed the ultra-structures of oligodendrocyte (white arrows) and myelin sheath. Lens: × 7500; Scale bar: 5 μm. **g** Representative images of olig2 and CC1 double immunostaining at 7 days post ICH. Lens: × 200; Scale bar: 100 μm. **h** Quantification of CC1^**+**^olig2^**+**^ cells, *n* = 6/group, * *p* < 0.01 versus ICH + vehicle, # *p* < 0.01 versus ICH + CeNP, **^** *p* < 0.05 versus ICH + vehicle+PLX3397. **i** Representative images of EdU and CC1 double immunostaining at 7 days post ICH. Lens: × 200; Scale bar: 100 μm. **j** Quantification of EdU^**+**^ CC1^**+**^ cells, *n* = 6/group, * *p* < 0.05 versus ICH + vehicle, # *p* < 0.05 versus ICH + CeNP, **^** *p* < 0.05 versus ICH + vehicle+PLX3397

Previous studies demonstrated that M2 microglia could promote oligodendrocyte differentiation, which was essential for remyelination in the CNS [12, 32]. PLX3397 (an inhibitor of colony-stimulating factor 1 receptor) was administrated to deplete microglia to investigate whether PEG-CeNP promotes remyelination in a microglia-dependent manner (Fig. 6a). The flow cytometry (Fig. 6b) and immunofluorescence staining (Fig. 6c) results showed that PLX3397 achieved good depletion efficacy in both the sham (Fig. 6d, mean ± SEM: 136 ± 8.23 versus 37 ± 2.59, *p* < 0.01 versus sham) and ICH mice (Fig. 6e, mean ± SEM: 884 ± 34.08 versus 324 ± 31.00, *p* < 0.01 versus ICH), respectively. The electron micrograph showed the essential role of mature oligodendrocyte in myelination (Fig. 6f).

The CC-1 and Olig2 double immunostaining images showed the quantity of mature oligodendrocytes (CC-1^**+**^ Olig2^**+**^) in the peri-hematoma region at 7 days post ICH (Fig. 6g). Mature oligodendrocytes were significantly increased in PEG-CeNP-treated mice (Fig. 6h, mean ± SEM: 256 ± 12.16 (ICH + CeNP) versus 209 ± 8.99 (ICH + vehicle), *p* < 0.01). Meanwhile, the number of CC-1^**+**^ Olig2^**+**^ cells was significantly decreased after PLX3397 administration (Fig. 6h, mean ± SEM: 86 ± 9.60 (ICH + vehicle + PLX3397) versus 209 ± 8.99 (ICH + vehicle), *p* < 0.01). PLX3397 administration also decreases the number of oligodendrocyte-lineage cells (Olig2^**+**^ cells, Figure S5A). Interestingly, we found that the number of mature oligodendrocytes was moderately enhanced in ICH + CeNP + PLX3397-treated mice when compared with the ICH + vehicle + PLX3397 group (Fig. 6h, mean ± SEM: 142 ± 4.89 (ICH + CeNP + PLX3397) versus 86 ± 9.60 (ICH + vehicle + PLX3397), *p* < 0.05).

In order to label proliferating cells, mice were intraperitoneally injected with a thymidine analog (EdU). The EdU and CC-1 double immunostaining images showed that the newly differentiated mature oligodendrocytes were significantly increased in ICH + CeNP-treated mice at 7 days after ICH (Fig. 6i, j, mean ± SEM: 50.83 ± 5.72 (ICH + vehicle) versus 70 ± 6.91 (ICH + CeNP), *p* < 0.05). PLX3397 administration decreased the number of proliferating cells (EdU^+^ cells, Figure S5B). Meanwhile, PEG-CeNP treatment also moderately promoted the expression of EdU^+^ and CC-1^+^ cells in ICH + CeNP +PLX3397-treated mice compared to ICH + vehicle + PLX3397 group (Fig. 6i, j, mean ± SEM: 55.83 ± 5.31 (ICH + CeNP +PLX3397) versus 28.33 ± 6.91 (ICH + vehicle + PLX3397), *p* < 0.05).

In light of the results mentioned above, we assumed that PEG-CeNP promoted OPCs differentiation, partly in a microglia-dependent manner, and astrocytes might also participate in the pathophysiological process.

### ICH-induced A1 astrocyte alteration

**Fig. 7:**
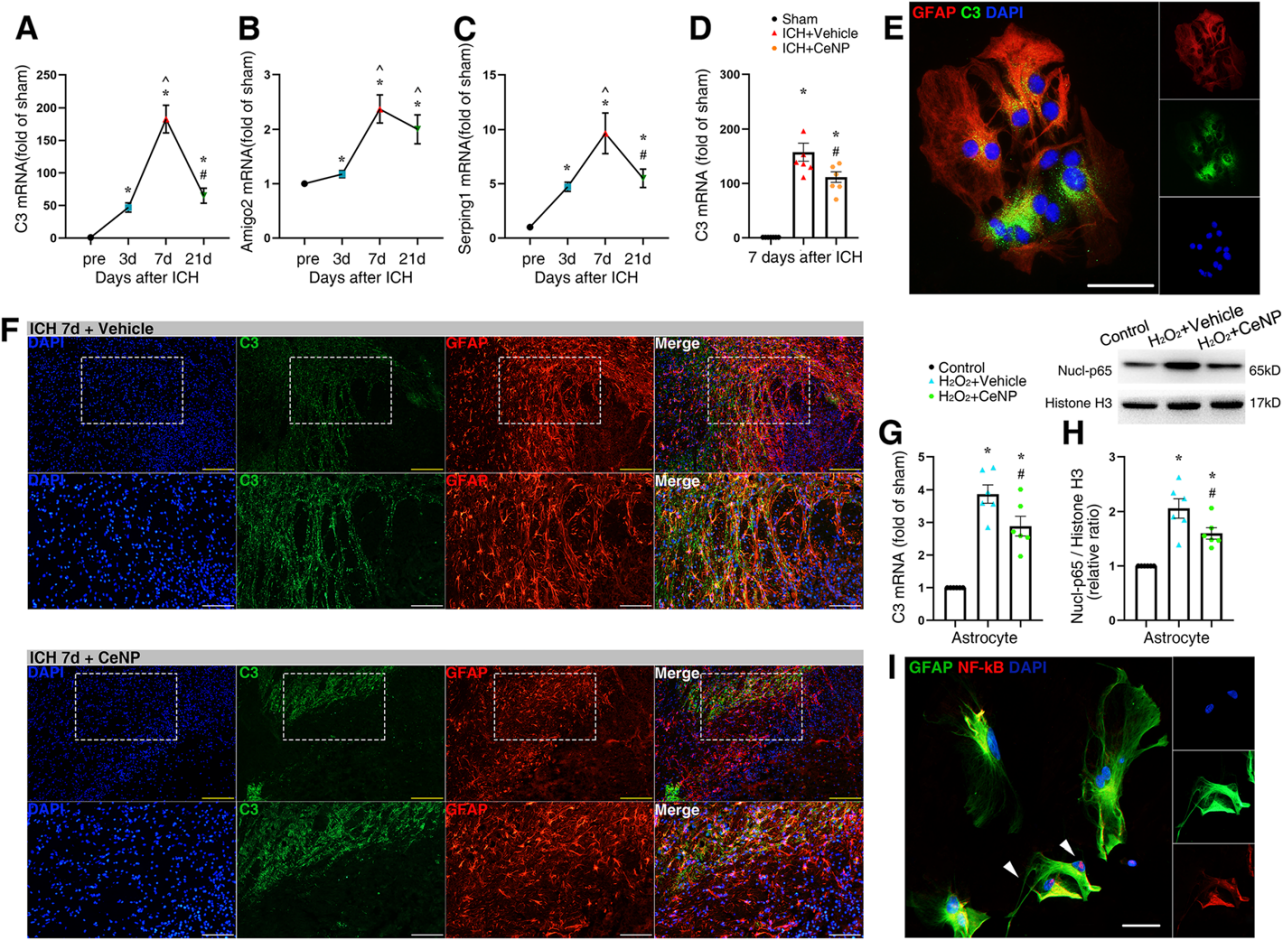
PEG-CeNP treatment protected against A1 astrocyte alteration after ICH. Representative RT-qPCR analysis of A1 astrocyte markers expression. **a** C3 mRNA expression. **b** Amigo2 mRNA expression. **c** Serping1 mRNA expression. *n* = 6/group, * *p* < 0.01 versus Sham. **^** *p* < 0.01 versus ICH 3d. # *p* < 0.01 versus ICH 7d. **d** C3 mRNA expression in PEG-CeNP-treated mice at 7 days post ICH. *n* = 6/group, * *p* < 0.01 versus Sham, # *p* < 0.01 versus ICH + vehicle. **e** Representative images of GFAP and C3 double immunostaining in cultured astrocytes (treated with H_2_O_2_). Lens: × 400; Scale bar: 50 μm. **f** Representative images of GFAP and C3 double immunostaining in brain sections at 7 days post ICH. Lens: × 100, × 200; Scale bar: 200 μm (yellow), 100 μm (white). **g** C3 mRNA expression in cultured astrocytes. *n* = 6/group, * *p* < 0.05 versus Control, # *p* < 0.05 versus H_2_O_2_ + vehicle. **h** Representative Western blot band showed the expression of nuclear NF-κB p65 in cultured astrocytes, *n* = 6/group, * *p* < 0.05 versus Control, # *p* < 0.05 versus H_2_O_2_ + vehicle. **i** Representative images of NF-κB p65 and GFAP double immunostaining in cultured astrocytes (treated with H_2_O_2_). Lens: × 400; Scale bar: 25 μm

As we know, A1 astrocytes lose most normal astrocytic functions and exert a neurotoxic function that rapidly devastate mature, differentiated oligodendrocytes [14]. To investigate the profile of astrocyte alteration during the ICH process, RT-PCR was used to examine the mRNA expression of A1 astrocyte markers in the brain tissue at 3, 7, and 21 days after ICH. As the most characteristic marker in A1 astrocytes, the mRNA expression of complement component 3 (C3) was significantly elevated (approximately 50–200 folds higher than sham) and peaked at 7 days after ICH induction (Fig. 7a, *p* < 0.01). In addition, ICH also induced the upregulation of Amigo2 and Serping1 mRNA expression (Fig. 7b, c, *p* < 0.01). Representative images of C3 and GFAP double staining at 3, 7, and 21 days post ICH were also in accordance with the tendency of C3 mRNA expression (Figure S6).

### PEG-CeNP treatment hindered A1 astrocyte alteration via inhibiting ROS-induced NF-κB p65 translocation

As shown in Fig. 7d, PEG-CeNP treatment resulted in the downregulation of C3 mRNA expression after ICH (*p* < 0.01 versus ICH + vehicle). Likewise, the C3 and GFAP double staining image also indicated that PEG-CeNP treatment significantly decreased A1 astrocyte expression at 7 days post ICH (Fig. 7f). Previous studies demonstrated that ROS might induce astrocyte activation [20], and A1 reactive astrocyte might be induced by NF-κB signaling [33, 34]. Hence, the cultured astrocytes were treated with H_2_O_2_ to investigate whether ROS could induce A1 reactive astrocyte directly. We found that H_2_O_2_ treatment strongly induced A1 astrocyte (Fig. 5e) compared to the control group (Figure S7). Simultaneously, the C3 mRNA expression also strongly increased in H_2_O_2_-treated astrocytes (Fig. 7g, *p* < 0.05 versus control), and PEG-CeNP treatment reversed the change (Fig. 7g, *p* < 0.05 versus H_2_O_2_ + vehicle). The western blot analysis showed that nuclear NF-κB p65 was significantly increased in astrocytes after H_2_O_2_ stimulation (Fig. 7h, *p* < 0.05 versus control). PEG-CeNP treatment inhibited the translocation of NF-κB p65 from the cytoplasm to the nucleus (Fig. 7h, *p* < 0.05 versus H_2_O_2_ + vehicle). Representative images of NF-κB p65 and GFAP double immunostaining in cultured astrocytes also indicated that H_2_O_2_ treatment resulted in the translocation of NF-κB p65 from the cytoplasm to the nucleus (Fig. 7i) compared to the control group (Figure S8).

### Astrocytes communicated with microglia via an astrocytic C3-microglial C3aR axis after ICH

**Fig. 8:**
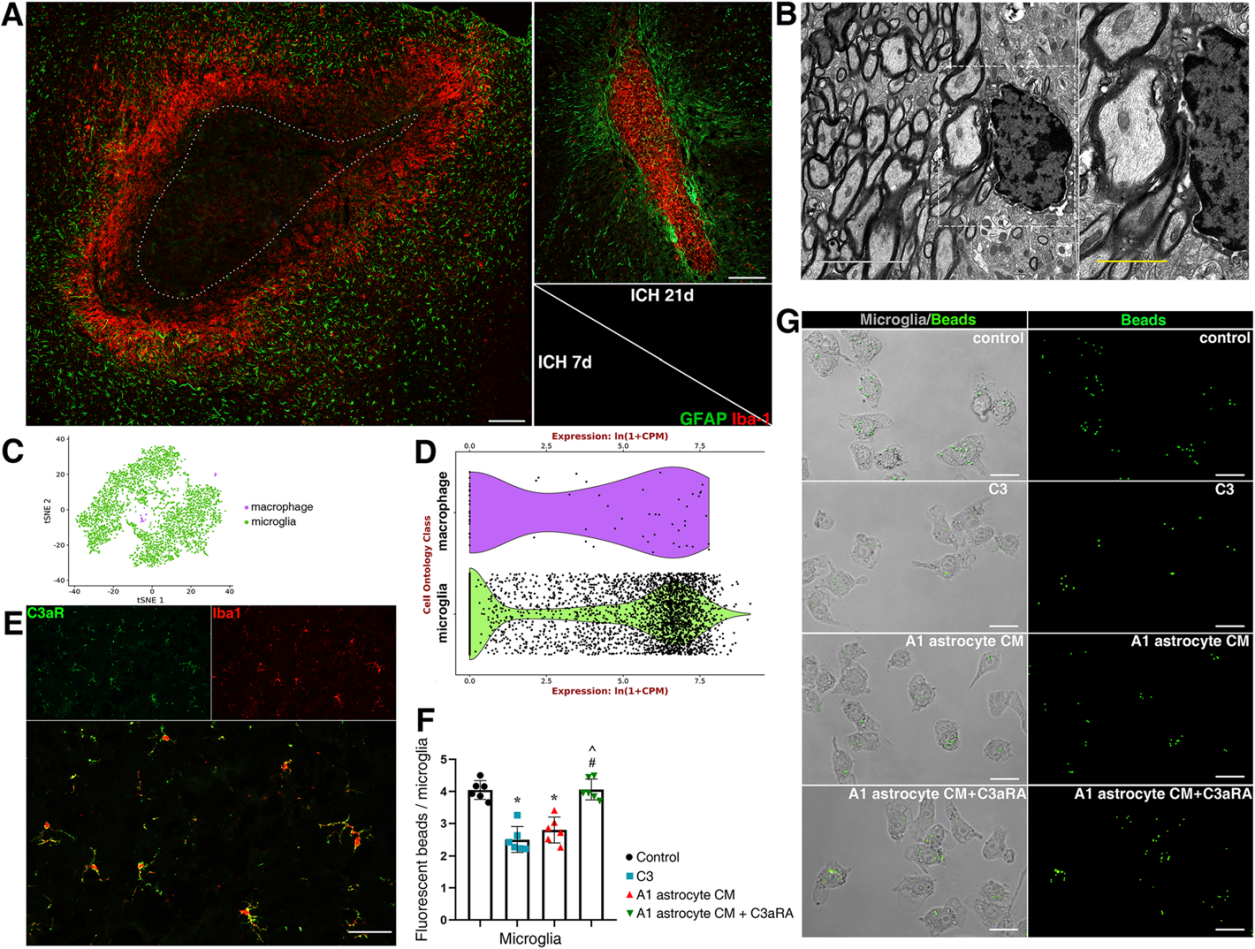
A1 astrocytes inhibited microglial phagocytosis of myelin debris via an astrocytic C3-microglial C3aR axis. **a** Representative images of GFAP and Iba1 double immunostaining in brain sections. Lens: × 100; Scale bar: 200 μm. **b** Representative TEM image showed microglia phagocytosis of myelin debris. Lens: × 7500, × 15,000; Scale bar: 5 μm (white), 2 μm (yellow). **c** tSNE plots of brain myeloid cells (Tabula Muris). **d** Expression of C3aR1 genes in microglia and macrophage (Tabula Muris). **e** Representative images of C3aR and Iba1 double immunostaining in brain sections. Lens: × 200; Scale bar: 50 μm. **f** Quantification of fluorescent beads in cultured microglia, * *p* < 0.01 versus Control, # *p* < 0.01 versus C3 treatment, **^** *p* < 0.01 versus A1 astrocyte CM treatment. **g** Representative images of fluorescent beads uptake in cultured microglia. Lens: × 400; Scale bar: 25 μm

Astrocyte and microglia have a unique bond and always coordinate their functions when the brain is perturbed [13]. Previous studies demonstrated that plenty of activated microglia were embedded in a GFAP-rich glial scar, and rapidly increased their phagocytic capacity to remove debris after CNS injury [13, 35]. The same phenomenon was also observed in the experimental ICH model (Fig. 8a). An astrocytic scar was formed to enclose the hematoma lesion and restricted the development of neuroinflammation. The embedded microglia exerted their phagocytic capacity to remove myelin debris, which can be seen in the electron micrograph (Fig. 8b). dMBP immunostaining was used to investigate whether PEG-CeNP treatment increases the microglial engulfment of myelin debris in vivo. As shown in Figure S9, PEG-CeNP treatment effectively reduced the percentage of dMBP area compared to ICH + vehicle at 3 days after ICH (*p* < 0.05, mean ± SEM: 6.07 ± 0.56% (ICH + vehicle) versus 4.30 ± 0.38% (ICH + CeNP)). Under the physiological condition, C3 could mediate the microglial engulfment of the redundant spines to ensure synaptic refinement via the C3/C3aR signaling pathway [29, 36]. As shown in Fig. 8c, d, the data from a single-cell transcriptome database indicated that the C3a receptor (C3aR) is strongly expressed in microglia (Tabula Muris). The immunostaining image of brain tissue cryosection also showed that C3aR was abundantly expressed in microglia (Fig. 8e). Since A1 astrocyte highly expressed C3, fluorescent beads were used to investigate whether astrocytes mediated microglial phagocytosis via an astrocytic C3-microglial C3aR axis (Fig. 8g). Microglia showed good phagocytosis in the control group, C3 and A1 astrocyte CM treatment strongly diminished the bead uptake (Fig. 8f, g, *p* < 0.01 versus control). However, C3aR antagonist (C3aRA, SB290157) administration could reverse the negative effects of C3 and A1 astrocyte CM in microglial phagocytosis (Fig. 8f, g, *p* < 0.01 versus C3 and A1 astrocyte CM). These results indicate that an astrocytic C3-microglial C3aR axis plays a crucial role in astrocyte-microglia crosstalk.

## Discussion

Since free radicals play a prominent role in the pathological process after ICH, a number of preclinical and clinical studies regard antioxidants as the therapeutic strategies of hemorrhagic stroke [1, 37]. Although plenty of preclinical studies exhibit good neuroprotection via scavenging ROS, there are still no effective drugs in the clinical treatment of ICH. Factors such as poor blood-brain barrier (BBB) permeability and inefficient free radical scavenging capacity may contribute to treatment failure [37]. CeNPs are known to possess potent-free radical scavenging activity. CeNPs have both SOD mimetic (eradicating superoxide anions and hydroxyl radicals) and catalase mimetic (eradicating hydrogen peroxide), which enable it to eradicate ROS repetitively. Previous studies demonstrated that CeNP treatment could protect against BBB disruption and neuronal death in several CNS disorders [23, 38]. However, whether CeNP treatment will benefit WMI is still contentious. ICH commonly occurs around the basal ganglia, which contains abundant white matter fibers (internal capsule). It indicates that WMI is dominant after ICH instead of gray matter injury. Hence, the present study is designed to investigate the putative therapeutic effects of CeNP on white matter repair via its ROS scavenging capacity in regulating microglial and astrocyte cells.

As we know, ROS is highly elevated in perihematomal white matter after ICH, which can oxidize protein, lipids, and nuclear material [15]. Oligodendrocyte-lineage cells (including OPCs and OLs) incorporate abundant lipids and lack essential glutathione and glutathione peroxidase. These specific characteristics make it highly vulnerable to oxidative stress and ultimately results in WMI [16]. The present study showed that ICH induced severe WMI, and PEG-CeNP treatment strongly reduced ROS levels in the brain tissue. In addition, PEG-CeNP treatment also improved the anatomical integrity of myelinated fibers and improved white matter integrity after ICH.

**Fig. 9:**
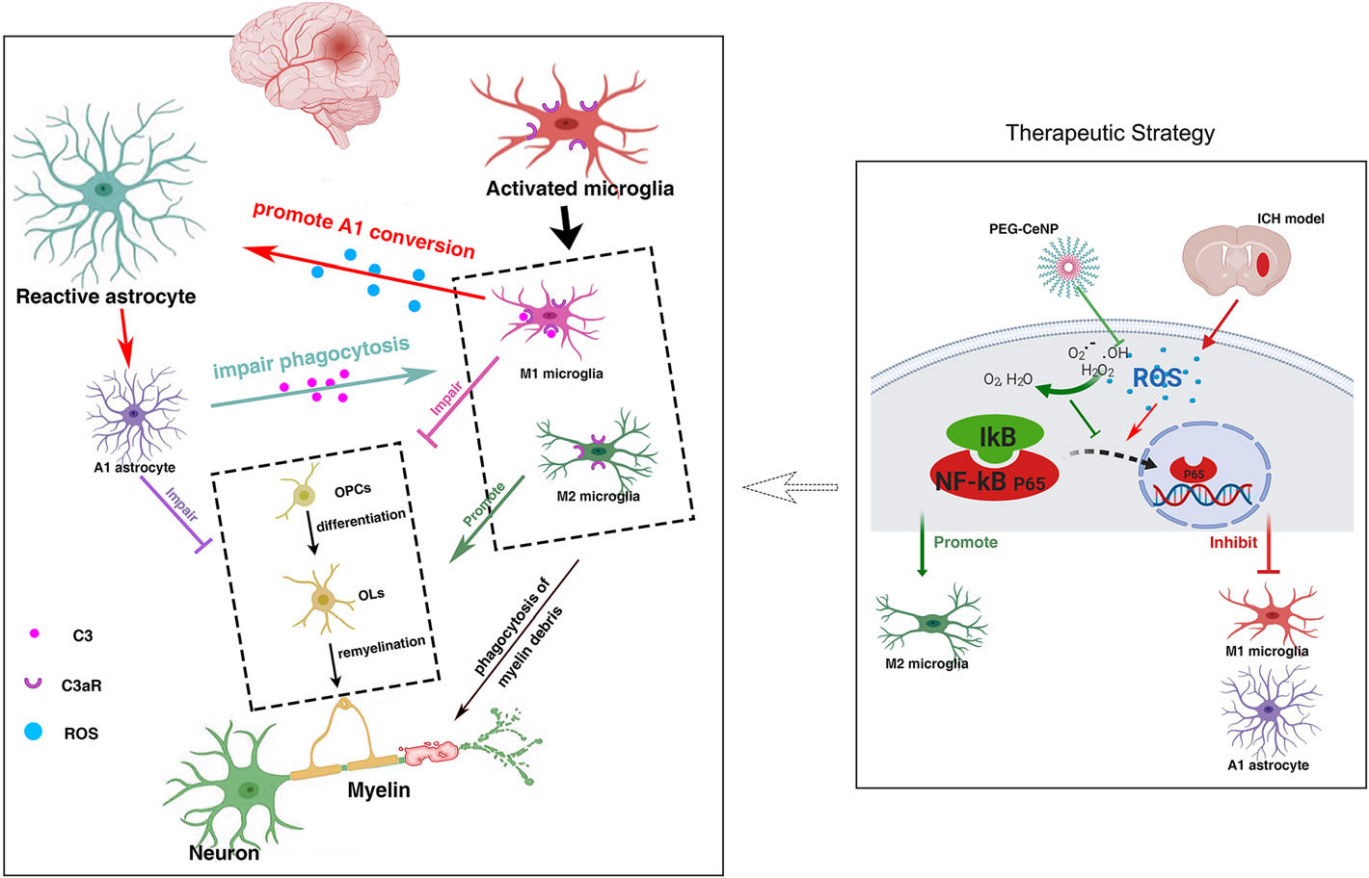
Possible pathogenesis and therapeutic strategy

Notably, ROS plays a prominent role in microglia activation and M1/M2 shift [17–19]. As the resident immune cell in the CNS, substantial evidence supports the crucial role of microglia in coordinating oligodendroglia lineage cell responses during remyelination. M2 microglia promote OPC proliferation and differentiation by releasing neurotrophic factors (IGF-1, VEGF, IL-13), and also strengthen the phagocytosis of myelin debris [11, 39]. In contrast, M1 microglia favor the production and release of cytokines that aggravate OPCs and OLs damage [40]. Hence, manipulation of microglia polarization can be an effective strategy to promote remyelination (Fig. 9). PEG-CeNP treatment likely promotes remyelination in a microglia-dependent manner.

Our data showed that ICH strongly induced microglia M1 polarization, which may further hinder OPC differentiation and cause demyelination. Bosetti et al. demonstrated that microglial ROS scavenging through genetic abrogation of NADPH oxidase attenuates M1 response and elevates M2 markers [19]. Consistent with the previous study, the results of the RT-PCR, immunofluorescence, and flow cytometry study revealed that PEG-CeNP treatment inhibited M1 polarization and promoted microglia toward M2 state.

As a prominent transcription factor, NF-κB is considered as a master inflammatory regulator, which is rapidly activated in the perihematomal region after ICH induction [15]. The NF-κB is a homo or heterodimer of the Rel family including 5 structural homologs: NF-κB1 (p50), NF-κB2 (p52), RelA (p65), RelB, and c-Rel. The p65/p50 dimer is the best-known inducer of an inflammatory response, which is fully functional in microglia [31]. Under normal conditions, IκB-α always binds with the p65/p50 dimer to inhibit its activity [18]. ROS induce the inactivation and degradation of IκB-α, which result in the translocation of p65/p50 dimer and then induce M1-polarized reaction [11, 31]. In the present study, we found that ICH strongly induced NF-κB p65 activation, while PEG-CeNP treatment reversed this. The results of western blot and immunofluorescence revealed that PEG-CeNP treatment hindered IκB-α degradation and the subsequent translocation of NF-κB p65 from the cytoplasm to the nucleus in microglia in vitro.

These results partly support our conjecture that PEG-CeNP treatment promoted remyelination via inhibiting ROS-induced NF-κB p65 translocation and modulating skewed microglia polarization. Afterward, an inhibitor of colony-stimulating factor 1 receptor-PLX3397 was administrated for microglia depletion. We found that PEG-CeNP treatment significantly enhanced the quantity of mature OLs, and such positive effects were substantially blocked after microglia depletion. Interestingly, we found that the number of mature OLs was slightly higher in ICH + CeNP + PLX3397-treated mice than in those treated with ICH + vehicle + PLX3397. Therefore, we considered the possibility that astrocytes participated in the aforementioned pathophysiological process.

It is known that astrocytes play a critical role in remyelination [14, 34]. Their basic functions are essential in maintaining the extracellular matrix where oligodendrocytes survive and remyelinate the CNS. However, astrocytes can be detrimental under some pathological conditions, including neuroinflammation and oxidative stress [20, 34]. Barres et al. revealed that neuroinflammation and ischemia resulted in 2 different phenotypes of reactive astrocytes, termed A1 and A2 astrocytes [14]. The A1 neurotoxic astrocytes lose normal astrocytic functions and induce the death of neurons, OLs, and OPCs. A study on multiple sclerosis also demonstrated that A1 reactive astrocytes aggravated demyelination via hindering OPC differentiation and damaging differentiated OLs [28]. The RT-PCR results in our study showed that C3 mRNA expression at 7 days after ICH was approximately 200 folds higher than in the sham group. The immunostaining images of C3 and GFAP were also in accordance with the tendency of the C3 mRNA expression. These results showed that ICH could strongly induce A1 neurotoxic astrocytes.

Notably, A1 reactive astrocytes might be strongly associated with NF-κB activation [14, 34]. Recent studies have shown that amyloid-β (Aβ) directly activated astrocytic NF-κB and C3 release, consistent with the high expression of C3 seen in patients with Alzheimer’s disease [33]. Moreover, the Aβ-activated microglia CM also induced A1 harmful astrocytes via the NF-κB signaling pathway [27]. In addition, ROS is known to be implicated in driving astrocyte reactivity and regulating NF-κB activation [20, 21]. In light of these results, we assumed that ROS might directly induce A1 astrocyte alteration via the NF-κB signaling pathway after ICH. Then, A1 astrocyte exhibits detrimental effects on OPC differentiation and maturation (Fig. 9). The present study displayed that PEG-CeNP treatment resulted in the downregulation of A1 astrocyte expression after ICH. H_2_O_2_ treatment in vitro caused prominent translocation of NF-κB into the nucleus and increased C3 mRNA expression. PEG-CeNP treatment hindered A1 astrocyte expression, both in vivo and in vitro. These results help to verify our conjectures.

Except for the enhancement of OPC proliferation and differentiation, the clearance of myelin debris is also critical for white matter repair. Myelin debris hinders new myelin sheath extension and the recruitment/differentiation of oligodendrocyte-lineage cells. Microglia has the potent phagocytic potential for the engulfment of myelin debris, which is also essential for remyelination. In this study, we first reported that plenty of activated microglia were embedded in the astrocyte scar and exerted phagocytic capacity to remove debris after ICH. Notably, the complement cascade plays a crucial role in promoting synapse elimination by microglia [13]. C3/C3aR signaling is implicated in mediating microglial engulfment of redundant spines to ensure synaptic refinement under the physiological condition [29, 36]. The present study has displayed that A1 astrocytes highly expressed C3 both in vivo and in vitro. Meanwhile, C3aR was also abundantly expressed in microglia. A1 astrocytes may likely participate in mediating microglial engulfment of myelin debris via an astrocytic C3-microglial C3aR axis. As expected, the in vitro study indicated C3 and A1 astrocyte CM treatment strongly diminished fluorescent bead uptake. The negative effects of C3 and A1 astrocyte CM in microglial phagocytosis were substantially reversed after C3aR antagonist administration. The electron micrograph also indicated that microglia exerted their phagocytic capacity to remove myelin debris in perihematomal region. Namely, astrocytes could decrease microglial engulfment of myelin debris via an astrocytic C3-microglial C3aR axis after ICH (Fig. 9). Therefore, it is of great value to inhibit A1 astrocyte alteration in the therapeutic strategy for ICH.

Astrocytes and microglia have a unique bond and always respond as one unit when the brain is perturbed [13]. Microglia-astrocyte crosstalk is of burgeoning interest in numerous studies of CNS diseases. Recent work has illuminated that microglia induce A1 reactive astrocyte via secreting IL-1α, C1q, TNF after *lipopolysaccharide* stimulation in the CNS [14]. Moreover, astrocyte-derived IL-33 is implicated in regulating microglia-associated synapse pruning and increasing chemokine expression in microglia [13, 41]. In light of these previous studies, the results mentioned above in our study further replenish the feedback mechanism from astrocytes to microglia.

However, there remain some conjectures that have yet to be verified. As mentioned earlier, oligodendrocyte-lineage cells are rich in lipids, making them highly vulnerable to oxidative stress. The present study does not explore whether PEG-CeNP treatment directly promotes oligodendrocyte-lineage cell survival by eradicating intracellular ROS. In addition, the activated microglia are featured in producing and releasing abundant ROS to kill bacteria or cause neurotoxicity [42]. Since the present study has proved that ROS can directly induce A1 astrocyte, microglia may likely exacerbate A1 astrocyte alteration via releasing ROS (Fig. 9). Moreover, the expansion of the hematoma area can lead to secondary brain damage such as oxidative stress and inflammation and so on. The present study does not verify whether PEG-CeNP treatment can attenuate hematoma size. It is crucial and meaningful to investigate the relationship between microglia and hematoma . It is worth noting that astrocytes also play a vital role in the phagocytosis of synapses and clearance of debris. In diseases with severe myelin injury, including multiple sclerosis, and subacute ischemia, myelin debris is taken up by astrocytes through receptor-mediated endocytosis [43]. Hence, the role of astrocyte in the clearance of myelin debris and hematoma after ICH should be investigated in future studies.

## Conclusion

In summary, the present study has uncovered a new mechanism in WMI after ICH. Namely, ICH induces M1 microglia and A1 astrocyte through ROS-induced NF-κB p65 translocation that hinders OPC differentiation and maturation. Subsequently, A1 astrocytes inhibit microglial engulfment of myelin debris via an astrocytic C3-microglial C3aR axis. PEG-CeNP treatment inhibits ROS-induced NF-κB p65 translocation in both microglia and astrocytes and ultimately promotes remyelination. Such findings unveil a novel therapeutic strategy for white matter repair after ICH. Future studies should regard astrocytes and microglia as a functional unit that has the potential to handle the complicated biological processes in new ways.

## Supplementary Information

**Additional file 1: Figure S1.** Sample size and grouping information. **Figure S2.** Colloidal stability of PEG-CeNPs and normal CeNPs. **Figure S3.** Representative images of CD1632/Iba1 and CD206/Iba1 double immunostaining at 7 days post ICH. Lens: 200x; Scale bar: 100 μm. **Figure S4.** Representative images of CD1632/Iba1 and CD206/Iba1 double immunostaining at 21 days post ICH. Lens: 200x; Scale bar: 100 μm. **Figure S5.** Representative images of olig2+ cells and EdU+ cells at 7 days post ICH. * p < 0.01 versus ICH + vehicle, # p < 0.01 versus ICH + CeNP, Lens: 200x; Scale bar: 100 μm. **Figure S6.** Representative images of GFAP and C3 double immunostaining in brain sections at 7 days post ICH. Lens: 200x; Scale bar: 100 μm. **Figure S7.** Representative images of GFAP and C3 double immunostaining in cultured astrocytes (control group). Lens: 200x; Scale bar: 50 μm. **Figure S8.** Representative images of NF-κB p65 and GFAP double immunostaining in cultured astrocytes (control group). Lens: 400x; Scale bar: 25 μm. **Figure S9.** Representative images of dMBP immunostaining at 3 days post ICH. # *p* < 0.05 versus ICH + vehicle. Lens: 100x, 200x; Scale bar: 200 μm (yellow), 100 μm (white).

## Data Availability

All raw data used in this manuscript are available on reasonable request

## Abbreviations

BBB: Blood-brain barrier
CeNP: Ceria nanoparticles
CNS: Central nervous system
CM: Conditioned medium
C3: Complement component 3
C3aR: C3a receptor
C3aRA: C3aR antagonist
DHE: Dihydroethidium
ICH: Intracerebral hemorrhage
OPCs: Oligodendrocyte progenitor cells
OLs: Oligodendrocytes
PEG-CeNPs: Polyethylene glycol-CeNPs
PDL: Poly-d-lysine
PVDF: Polyvinylidene fluoride
ROS: Reactive oxygen species
SOD: Superoxide dismutase
SEM: Standard error of the mean
TEM: Transmission electron microscopy
