# Supplementary material for: Ceria nanoparticles ameliorate white matter injury after intracerebral hemorrhage: microglia-astrocyte involvement in remyelination
Source: J Neuroinflammation. 2021 Feb 15;18:43. doi: 10.1186/s12974-021-02101-6 (PMC7883579; doi:10.1186/s12974-021-02101-6)
Supplement: Supplementary file 1 — Additional file 1: Figure S1. Sample size and grouping information. Figure S2. Colloidal stability of PEG-CeNPs and normal CeNPs. Figure S3. Representative images of CD1632/Iba1 and CD206/Iba1 double immunostaining at 7 days post ICH. Lens: 200x; Scale bar: 100 μm. Figure S4. Representative images of CD1632/Iba1 and CD206/Iba1 double immunostaining at 21 days post ICH. Lens: 200x; Scale bar: 100 μm. Figure S5. Representative images of olig2+ cells and EdU+ cells at 7 days post ICH. * p < 0.01 versus ICH + vehicle, # p < 0.01 versus ICH + CeNP, Lens: 200x; Scale bar: 100 μm. Figure S6. Representative images of GFAP and C3 double immunostaining in brain sections at 7 days post ICH. Lens: 200x; Scale bar: 100 μm. Figure S7. Representative images of GFAP and C3 double immunostaining in cultured astrocytes (control group). Lens: 200x; Scale bar: 50 μm. Figure S8. Representative images of NF-κB p65 and GFAP double immunostaining in cultured astrocytes (control group). Lens: 400x; Scale bar: 25 μm. Figure S9. Representative images of dMBP immunostaining at 3 days post ICH. # p < 0.05 versus ICH + vehicle. Lens: 100x, 200x; Scale bar: 200 μm (yellow), 100 μm (white). [file 12974_2021_2101_MOESM1_ESM.docx]

**Supplementary materials**


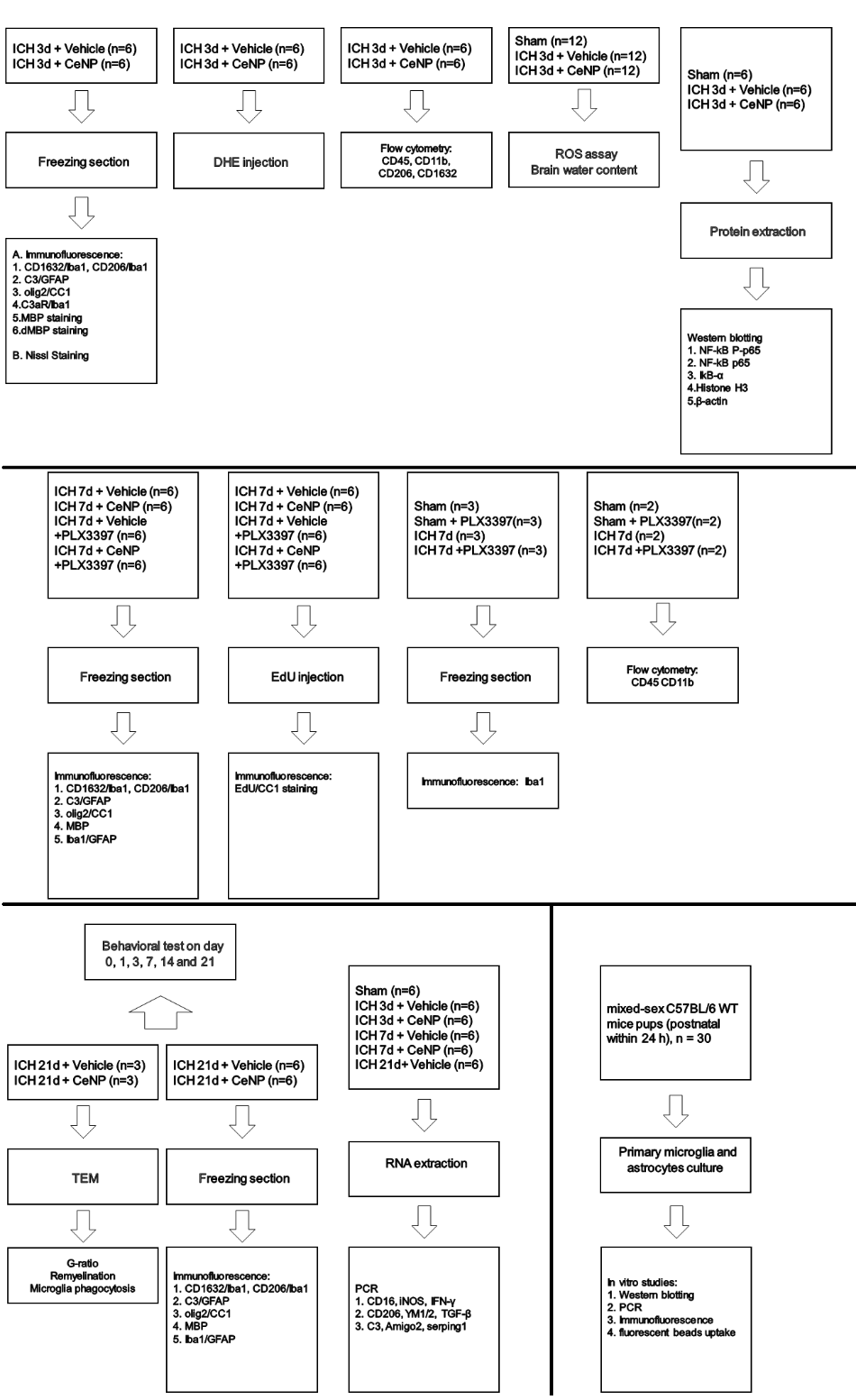


**FigureS1**

Sample size and grouping information


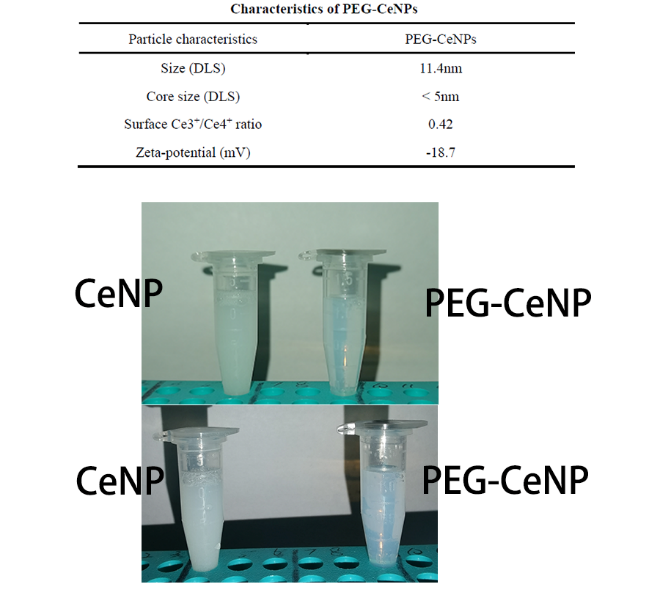


**FigureS2**

Colloidal stability of PEG-CeNPs and normal CeNPs


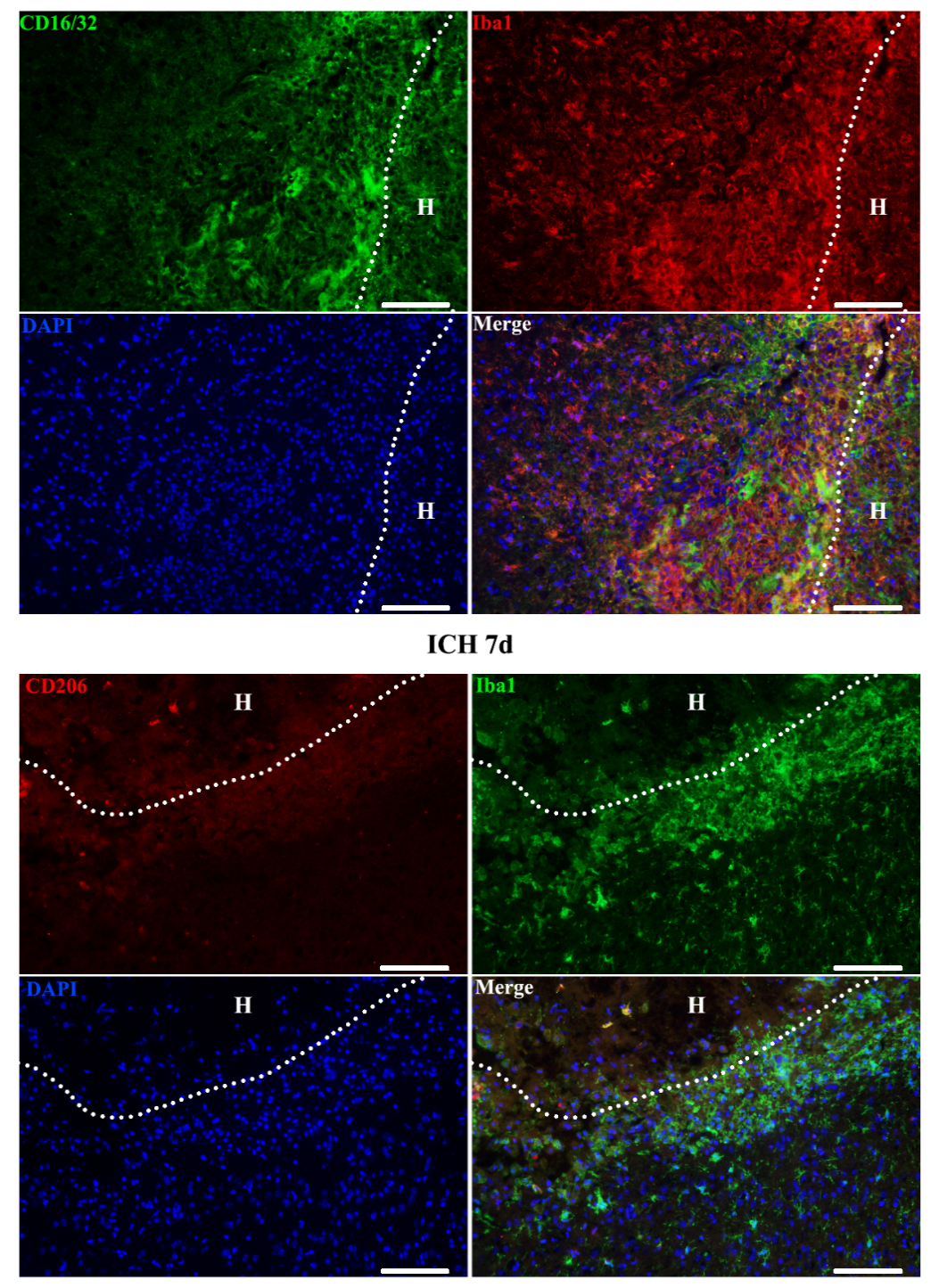


**FigureS3**

Representative images of CD1632/Iba1 and CD206/Iba1 double immunostaining at 7 days post ICH. Lens: 200x; Scale bar: 100μm.


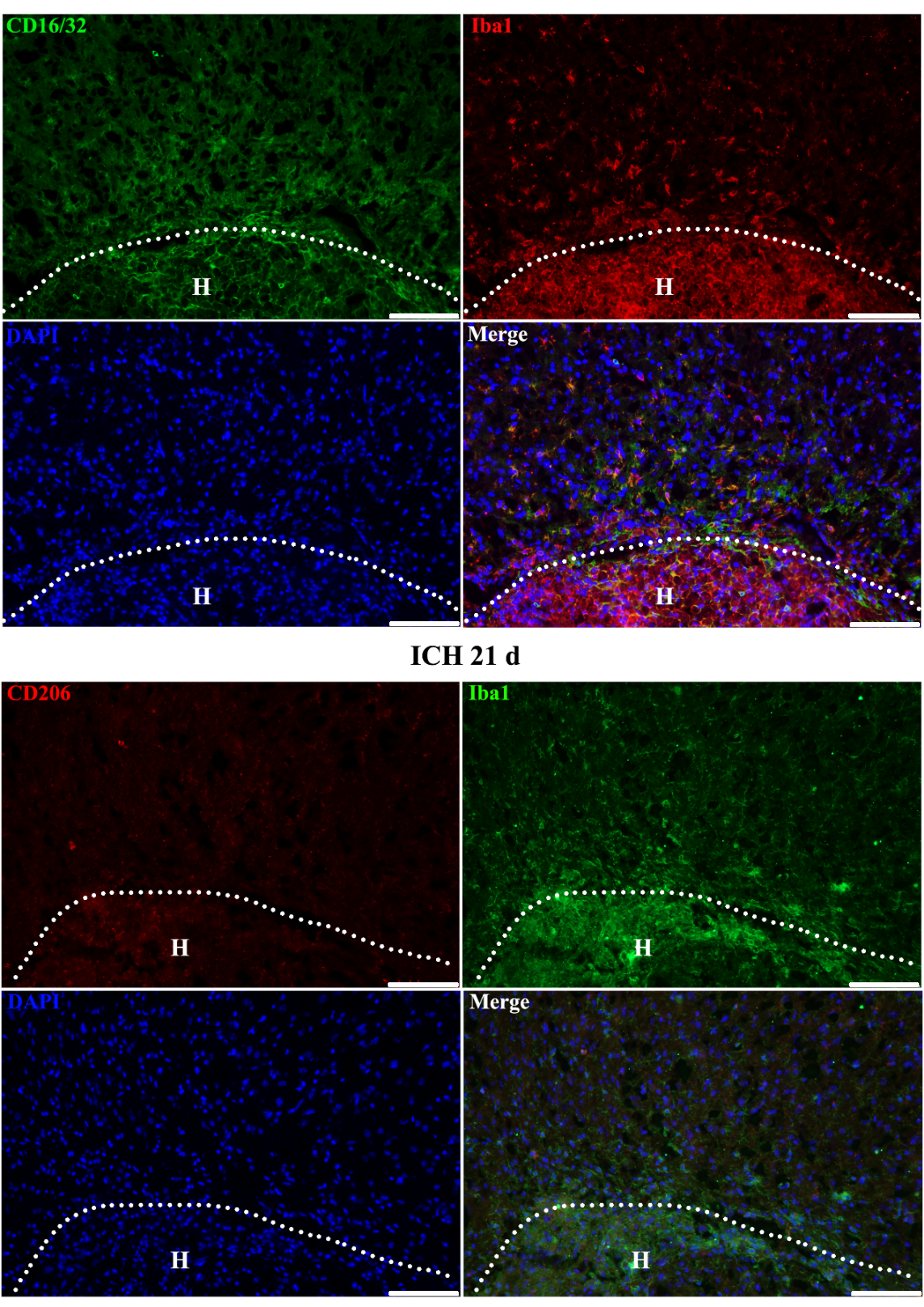


**FigureS4**

Representative images of CD1632/Iba1 and CD206/Iba1 double immunostaining at 21 days post ICH. Lens:200x; Scale bar: 100μm.


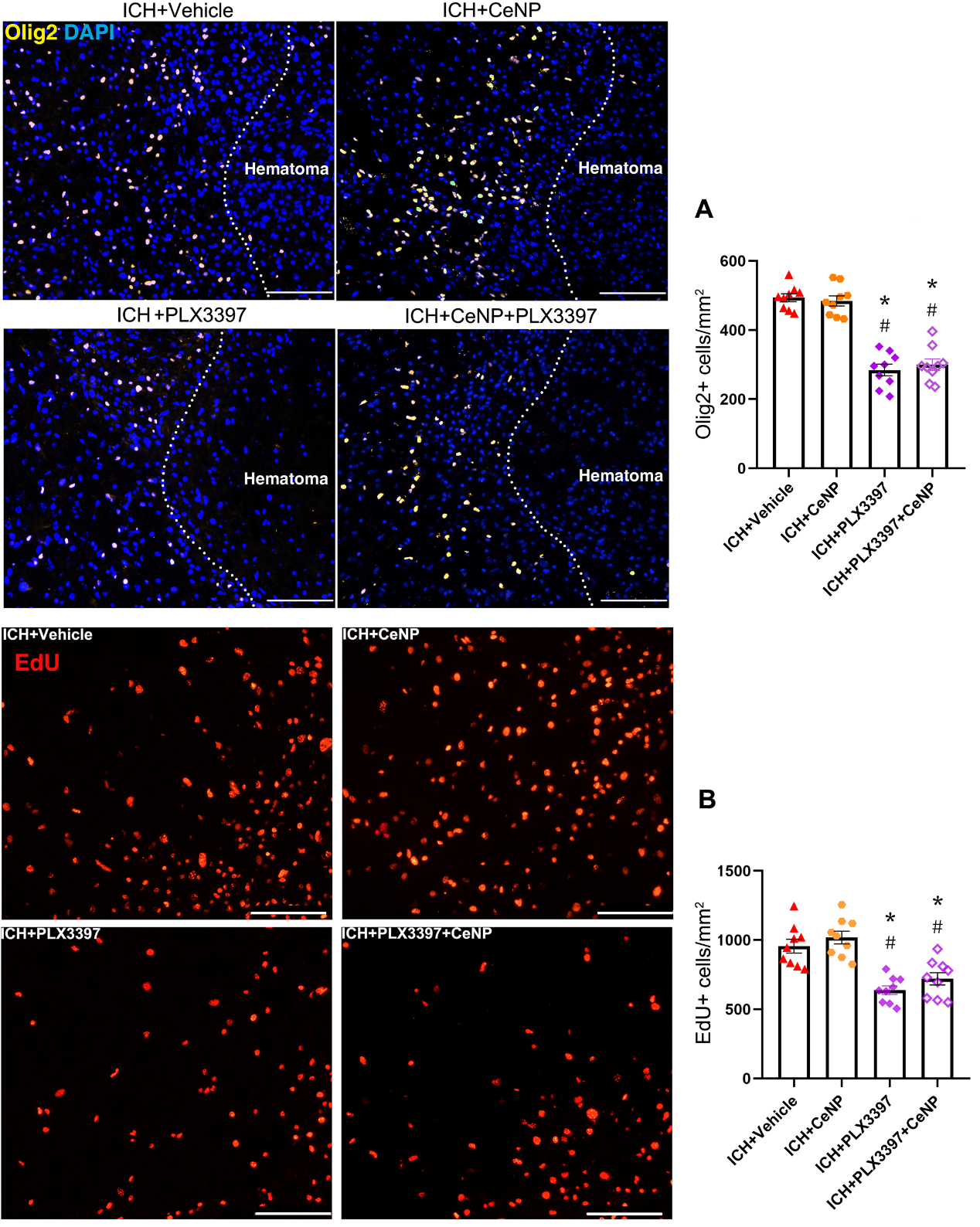


**FigureS5**

Representative images of olig2^+^ cells and EdU^+^ cells at 7 days post ICH. * *p* < 0.01 versus ICH + Vehicle, # *p* < 0.01 versus ICH + CeNP, Lens:200x; Scale bar: 100μm.


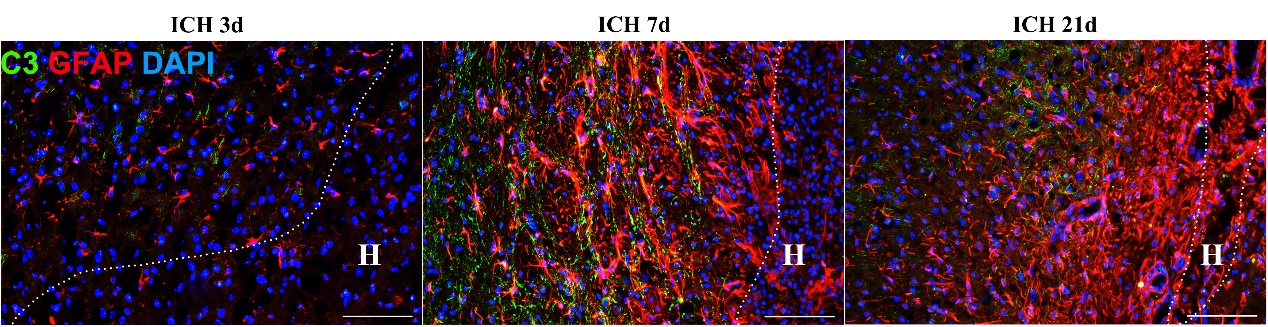


**FigureS6**

Representative images of GFAP and C3 double immunostaining in brain sections at 7 days post ICH. Lens: 200x; Scale bar: 100μm.


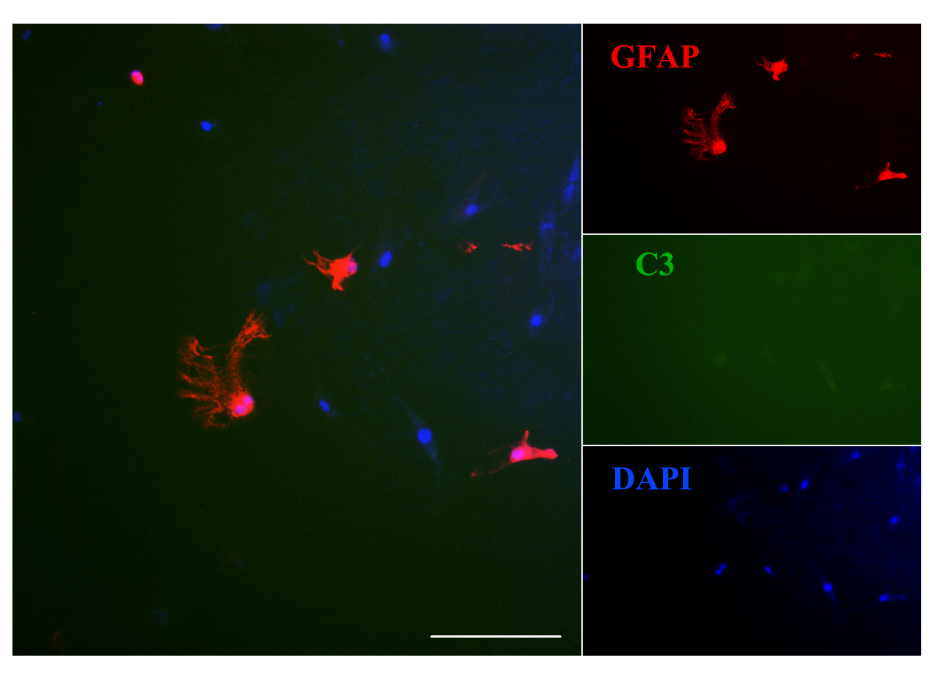


**FigureS7**

Representative images of GFAP and C3 double immunostaining in cultured astrocytes (control group). Lens: 200x; Scale bar: 50μm.


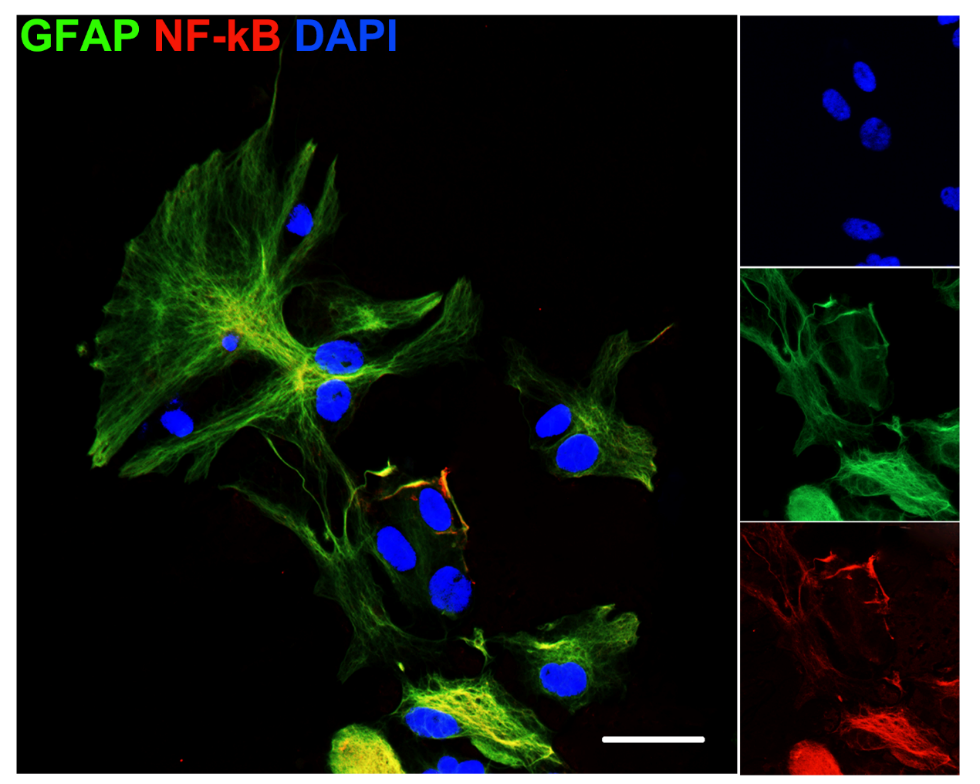


**FigureS8**

Representative images of NF-κB p65 and GFAP double immunostaining in cultured astrocytes (control group). Lens: 400x; Scale bar: 25μm.


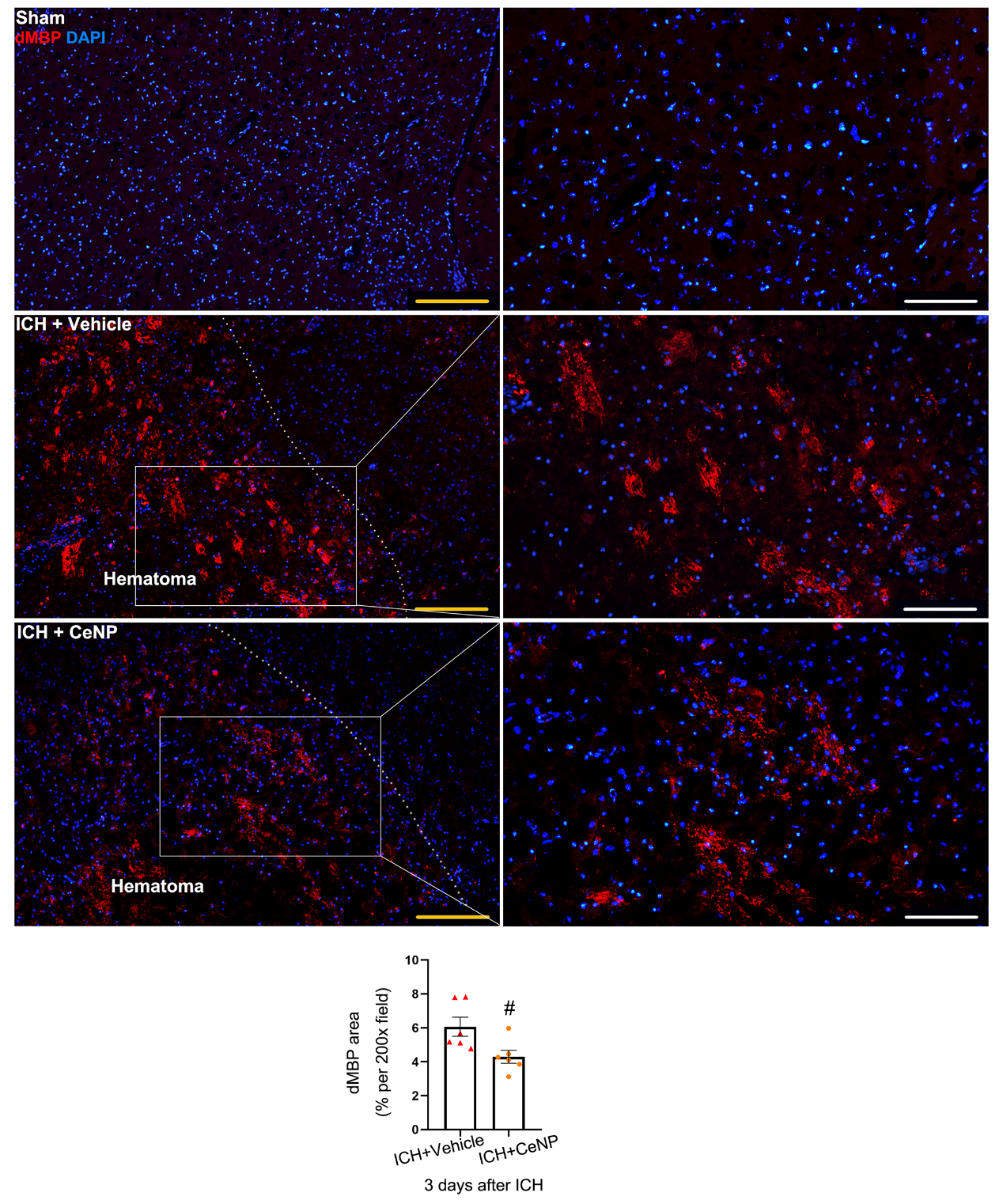


**FigureS9**

Representative images of dMBP immunostaining at 3 days post ICH. # *p* < 0.05 versus ICH + vehicle. Lens: 100x, 200x; Scale bar: 200μm (yellow), 100μm (white).
